# Radiation exposure and clinical validation of autosegmentation models for the supraventricular cardiac conduction system in breast cancer radiotherapy: an institutional perspective

**DOI:** 10.3389/fonc.2026.1734696

**Published:** 2026-01-29

**Authors:** Yanjun Zhang, Xiaochen Han, Zifeng Chi, Ziqi Zhao, Feifei Wang, Dan Liu, Ruoling Han

**Affiliations:** 1 Department of Radiation Oncology, The Fourth Hospital of Hebei Medical University, The Tumor Hospital of Hebei Province, Shijiazhuang, Hebei, China; 2 Department of Ultrasound, The Fourth Hospital of Hebei Medical University, Shijiazhuang, Hebei, China; 3 Department of Clinical Laboratory, The Fourth Hospital of Hebei Medical University, Shijiazhuang, China

**Keywords:** auto-segmentation, breast cancer, conduction nodes, dosimetry, IMRT, SAN

## Abstract

**Background:**

Radiation dose to cardiac conduction nodes may contribute to arrhythmia risks in breast cancer (BC) patients after radiotherapy, yet dosimetric evidence remains limited. This study aimed to evaluate doses to the sinoatrial (SAN) and atrioventricular nodes (AVN) in BC patients treated with intensity-modulated radiation therapy (IMRT) and to clinically validate a deep learning-based autosegmentation model for these structures.

**Methods:**

A retrospective analysis was conducted on 87 BC patients who underwent IMRT. Doses to the whole heart, four cardiac chambers, the SAN, and the AVN were evaluated and correlated. For autosegmentation, a convolutional neural network (CNN) was trained on 60 patients, validated on seven, and tested on 20. Segmentation accuracy was assessed using the Dice similarity coefficient (DSC), and dosimetric consistency was compared between automated and manual contours.

**Results:**

In right-sided BC patients, the SAN received the highest mean dose among cardiac substructures (5.43 Gray [Gy]) under a mean heart dose of 3.39 Gy. Both SAN and AVN doses showed strong correlations with right atrial (RA) dose (*R*^2^ for SAN: 0.63 in left- and right-sided cases; for AVN: 0.77 and 0.63, respectively). The autosegmentation model achieved DSCs of 0.83 for SAN and 0.75 for AVN, with no statistically significant dosimetric differences between autosegmented and manual contours.

**Conclusions:**

The SAN receives substantial irradiation in right-sided BC patients during IMRT, and RA dose strongly correlates with conduction node doses, suggesting its potential as a clinical surrogate. The CNN-based autosegmentation method enables accurate and efficient delineation of the SAN and AVN, facilitating reliable dosimetric assessment in clinical practice.

## Introduction

Radiation therapy is an important adjuvant treatment for breast cancer and can effectively improve local control and patient survival (1). However, radiation-induced heart disease (RIHD) has become a major factor affecting long-term survival (2, 3). Since RIHD is dose-dependent, dose optimization and constraints on the heart and its substructures during treatment planning are essential for effective risk prevention and management (4).

With the application of intensity-modulated radiation therapy (IMRT) and VMAT, the average radiation dose to the whole heart and key structures (such as the left ventricle) has been reduced (5, 6); however, the low-dose radiation volume of the heart has simultaneously increased (5, 7). Studies have shown that irradiation of cardiac conduction systems located in low-dose regions, such as the sinoatrial (SAN) and atrioventricular nodes (AVN), is associated with an increased risk of long-term arrhythmias and conduction disorders (8–10). Epidemiological data show that breast cancer patients receiving radiotherapy have a significantly increased risk of severe conduction disorders requiring pacemaker intervention (11, 12). However, there is a lack of dosimetric evidence linking radiation dose to the conduction system with clinical outcomes. The soft tissue contrast of the SAN and AVN in radiotherapy planning CT is low, the anatomical structures are complex, and they are susceptible to physiological motion artifacts. Manual delineation of the SAN and AVN is time-consuming, and differences between contouring operators make it difficult to meet the requirements of clinical research and precise radiotherapy planning.

In recent years, deep learning technology, especially the encoder–decoder architecture represented by U-shaped convolutional network (U-Net), has made a breakthrough in the field of medical image segmentation (13, 14). While improving the segmentation efficiency and consistency, it shows great potential in dealing with complex anatomical structures with low contrast (15).

Therefore, this study aims to evaluate the doses to the SAN and AVN in breast cancer patients receiving IMRT treatment. Specifically, the study seeks to: (1) explore whether the mean heart dose (MHD) and the dose to each cardiac chamber can serve as reliable alternative indicators for the dose to the conduction system, and (2) verify an automatic deep learning-based segmentation method for the cardiac conduction system, assessing its delineation robustness and dosimetric consistency, to provide a reliable tool for clinical dose–effect studies and radiotherapy plan optimization.

## Materials and methods

### Study population

Eighty-seven breast cancer (BC) patients who received postoperative radiotherapy with IMRT were retrospectively enrolled between October 2020 and October 2021. The median age was 55 years (interquartile range [IQR], 46–60), including 59 patients with left-sided BC and 28 patients with right-sided BC. During computed tomography (CT) simulation, patients were positioned supine on a breast board with arms raised and supported. Free-breathing scans were acquired with a slice thickness of 3 mm. All CT images were imported into the Pinnacle 8.0 treatment planning system for three-dimensional reconstruction. Patients were treated with conventionally fractionated, free-breathing IMRT using 6 MV photon beams via tangential fields. The planning target volume (PTV) received a total dose of 50.0–50.4 Gray (Gy) in 25–28 fractions. An additional boost dose of 10.0–10.2 Gy was administered to the tumor bed. Treatment plans were optimized according to the International Commission on Radiation Units (ICRU) guidelines to achieve a standardized dose distribution at the breast reference point. Dose constraints for organs at risk, including the heart, were applied following the Quantitative Analyses of Normal Tissue Effects in the Clinic (QUANTEC) recommendations (16).

### Delineation of cardiac conduction nodes

**Figure 1:**
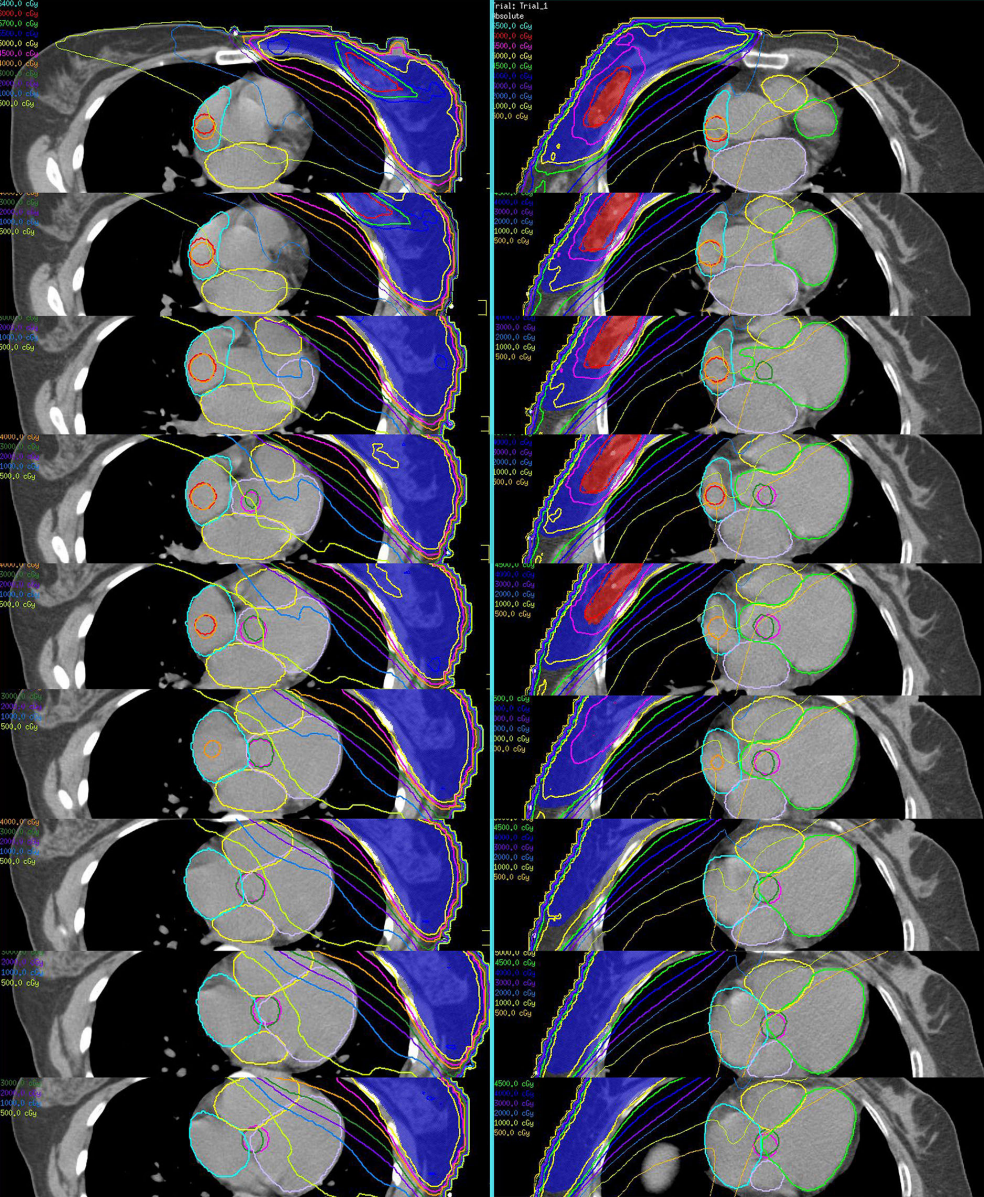
Dose distribution of conduction nodes in the left and right BC regions using IMRT.

According to previously published guidelines (17), the SAN and AVN were manually segmented on the pCT by a radiation therapist and an imaging physician, respectively, and reviewed by the chief physician. The SAN was delineated as a sphere with a diameter of 2.0 cm, tangent to the outer wall of the right atrium and centered at the level of the ascending aorta. The AVN was delineated as a sphere with a 2.0-cm diameter, centered at the junction of the four heart chambers and located 1.0 cm above the most inferior slice where the left atrium is visible (18). Concurrently, the right atrium (RA), right ventricle (RV), left atrium (LA), and left ventricle (LV) were manually delineated (19, 20). The two-dimensional dose distribution of the cardiac substructures is shown in **Figure 1**. For further details, please refer to the **Supplementary Material**.

### SAN and AVN autosegmentation model training

**Figure 2:**
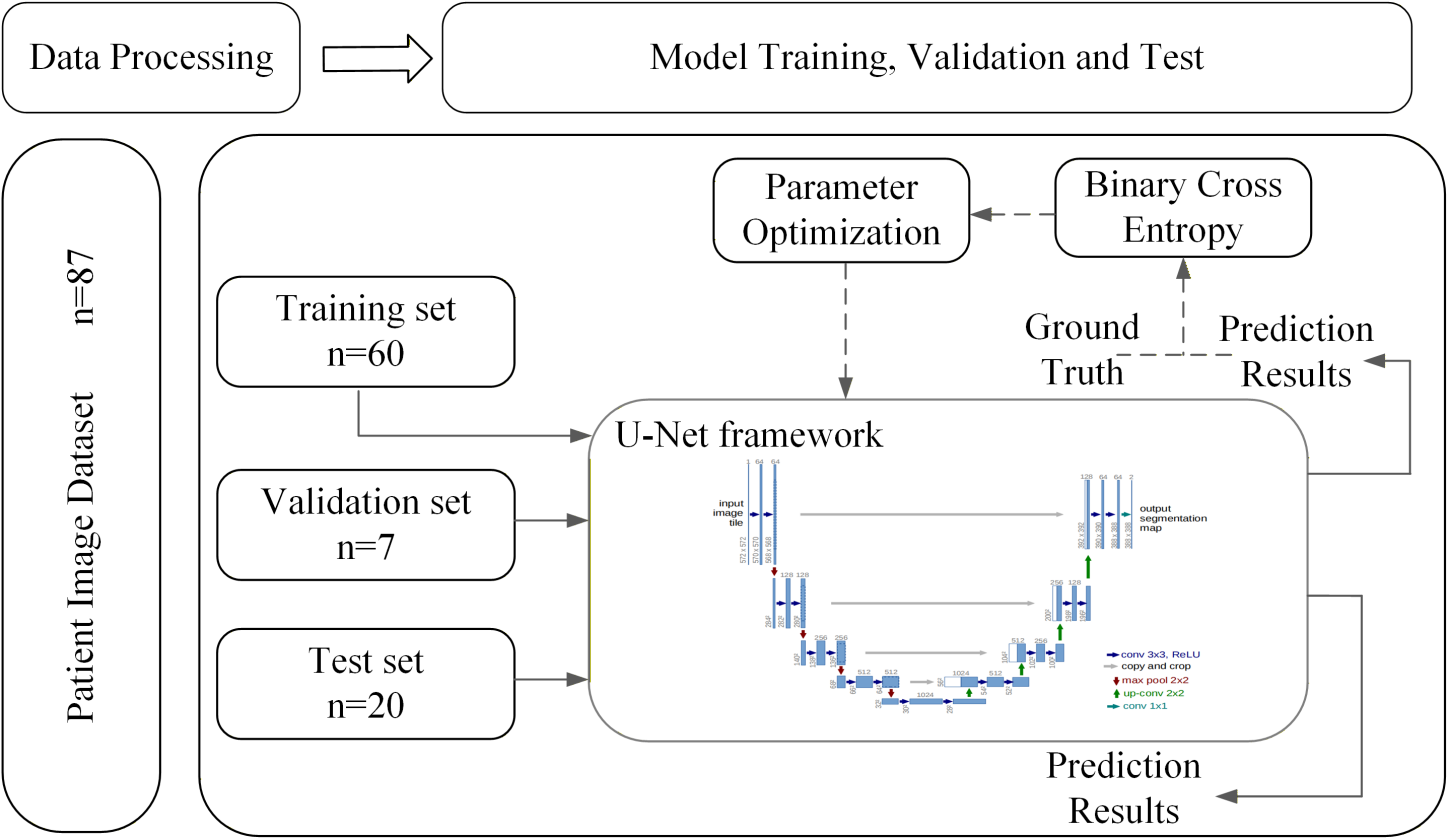
Workflow of the SAN and AVN autosegmentation model training.

To construct the automatic segmentation model for the SAN and AVN, this study utilized a two-dimensional U−Net architecture. The model was trained using noncontrast planning CT scans from 65 patients, along with the corresponding manual contours. All implementations were performed in Python 3.8.10 with the PyTorch 2.0.0 framework, using an NVIDIA TITAN RTX GPU (24 GB memory) for computation. To ensure robust evaluation and mitigate overfitting, the dataset was randomly partitioned into three subsets: training set (60 patients), validation set (seven patients), and test set (20 patients). The training parameters were as follows: (1) mode: unet_2d; (2) batch size: 6; (3) learning rate: 1*e*−4; (4) loss function: binary cross entropy loss; (5) max epochs: 100. The optimizer used was Adam. Data augmentation and early stopping were employed to improve generalization. The training and validation loss curves demonstrated stable convergence without signs of overfitting, indicating good learning stability of the model (**Figure 2**). For further details, please refer to the **Supplementary Material**.

### Statistical analysis

SPSS 27.0 software was used to analyze the parameters. Mean (*D*_mean_) and maximum doses (*D*_max_) of cardiac structures (WH, LV, LA, RV, RA, SAN, and AVN) were obtained from the DVH. Physical doses are reported in Gray. The data showed a nonnormal distribution; therefore, the median and IQR (p25–p75) were used for statistical description. The Mann–Whitney *U* test was applied for intergroup comparison (*p* < 0.05). The dose ratio of SAN/AVN to each cardiac structure was calculated. Spearman correlation analysis was used to evaluate correlations between doses (*r* > 0.7 was considered a strong correlation), and simple linear regression was applied to assess the predictive ability of each cardiac structure dose for the SAN/AVN dose (*R*^2^ > 0.6–0.7 was considered predictive). Bonferroni correction was applied to adjust significance levels (*p* < 0.01). For model residual analysis and *post hoc* power analysis, please refer to the **Supplementary Material**.

## Results

### Dose exposure and association of cardiac substructures

**Figure 3:**
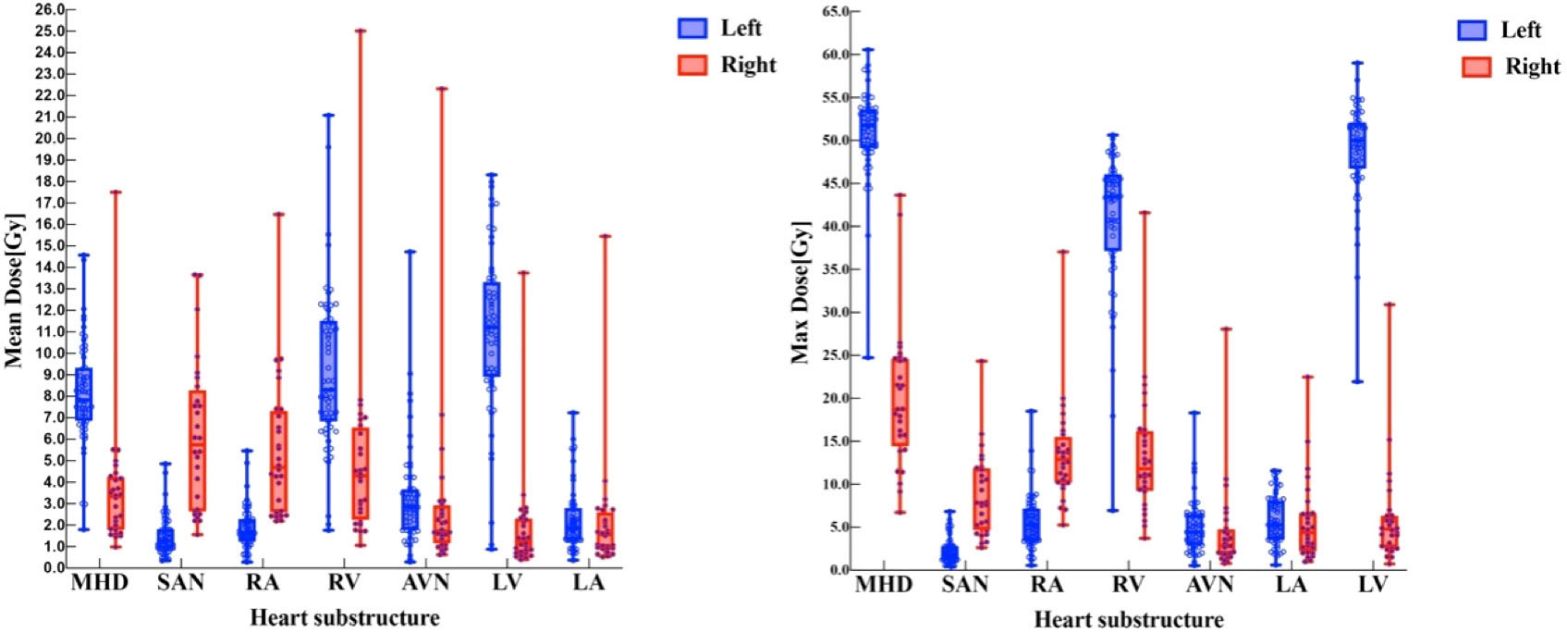
*D*_mean_ and *D*_max_ distributions of substructures in the left- and right-sided BC.

In right-sided BC patients, the MHD was 3.39 Gy (IQR, 1.82–4.28 Gy). The SAN was the substructure with the highest *D*_mean_ of 5.43 Gy (IQR, 2.73–7.78 Gy), followed by the RA, with a *D*_mean_ of 4.59 Gy (IQR, 2.66–7.08 Gy). Of note, 60.7% of the patients received more than 5.0 Gy to the SAN, while 46.4% received more than 5.0 Gy to the RA. The AVN received a *D*_mean_ of 1.80 Gy (IQR, 1.37–3.00 Gy), and 10.7% of patients received more than 5.0 Gy. In left-sided BC, the *D*_mean_ was 1.29 Gy (IQR, 0.89–1.82 Gy) for the SAN and 2.86 Gy (IQR, 1.77–3.67 Gy) for the AVN, with 11.9% of patients receiving more than 5.0 Gy to the AVN. The LV was the most exposed substructure, with a *D*_mean_ of 11.2 Gy (IQR, 9.1–13.32 Gy). The MHD was 8.19 Gy (IQR, 6.87–9.34 Gy) (**Figure 3**; **Table 1**).

**Table 1:** Dose distribution of substructures in right- and left-sided BC patients.

Structures	Right-sided BC			Left-sided BC		
	*D*_mean_	*D*_max_	*D*_mean_	*D*_mean_	*D*_max_	*D*_mean_
	Median (IQR)	Median (IQR)	> 5.0 Gy (%)	Median (IQR)	Median (IQR)	> 5.0 Gy (%)
Heart	3.39 (1.82–4.28)	18.76 (15.66–24.64)	14.3 (4/28)	8.19 (6.87–9.34)	51.73 (49.32–53.67)	96.6 (57/59)
SAN	5.43 (2.73–7.78)	7.85 (5.24–11.86)	60.7 (17/28)	1.29 (0.89–1.82)	1.78 (1.22–2.80)	0 (0/59)
AVN	1.80 (1.37–3.0)	2.85 (1.89–4.46)	10.7 (3/28)	2.86 (1.77–3.67)	4.45 (2.89–6.46)	11.9 (7/59)
RA	4.59 (2.66–7.08)	12.60 (10.25–14.61)	46.4 (13/28)	1.65 (1.24–2.3)	5.25 (3.41–7.13)	1.7 (1/59)
RV	4.40 (2.6–6.65)	11.19 (9.41–15.68)	35.7 (10/28)	8.70 (6.98–11.52)	43.48 (38.89–45.84)	93.22 (55/59)
LA	1.62 (0.94–2.25)	4.04 (2.65–6.62)	3.6 (1/28)	1.87 (1.32–2.54)	5.33 (3.69–8.08)	8.47 (5/59)
LV	1.38 (0.77–2.34)	4.87 (2.80–6.36)	3.6 (1/28)	11.2 (9.1–13.32)	50.02 (47.21–52.11)	94.92 (56/59)

For the SAN, all node–structure (N/S) dose ratios were > 1 in right-side BC and < 1 in left-side BC. This indicates that the SAN irradiation dose is higher in right-side BC than in the other substructures, whereas the opposite is true in left-side BC. Correlation analysis showed that the *D*_mean_ of SAN had the strongest correlation with the *D*_mean_ of the RA (Spearman correlation coefficient *r*: left = 0.80, right = 0.93). The *D*_mean_ of the AVN also showed the highest correlation with the *D*_mean_ of the RA dose in left-sided breast cancer (*r* = 0.81) (**Table 2**); this relationship was further validated by linear regression analysis. For the SAN, the coefficient of determination (*R*^2^) between its dose and the RA dose was 0.63 in both left- and right-sided patients. For the AVN, the *R*^2^ value between its dose was 0.77 in left-sided patients and 0.63 in right-sided patients (**Table 2**). *Post hoc* power analysis demonstrated adequate statistical power for detecting the key correlations and regression relationships described above, **Supplementary Material**.

**Table 2:** Association and relationship between *D*_mean_ and the SAN, AVN, and heart chambers.

Node	Structure	Left-sided BC					Right-sided BC				
		Ratio	Correlation		Liner regression		Ratio	Correlation		Liner regression	
		N/S	*r*	*p*-value	*R* ^2^	*p*-value	N/S	*r*	*p*-value	*R* ^2^	*p*-value
SAN	MHD	0.19	0.36	0.005^a^	0.13	0.0052^a^	1.89	0.77	< 0.0001^a,b^	0.47	< 0.0001^a,b^
	LA	0.68	0.53	< 0.0001^a,b^	0.18	0.0009^a,b^	2.89	0.58	0.0012^a,b^	0.59	< 0.0001^a,b^
	LV	0.14	0.22	0.099	0.05	0.078	3.24	0.61	0.001^a,b^	0.25	< 0.0001^a^
	RA	0.82	0.80	< 0.0001^a,b^	0.63	< 0.0001^a,b^	1.11	0.93	< 0.0001^a,b^	0.63	< 0.0001^a,b^
	RV	0.17	0.44	0.0005^a,b^	0.15	0.002^a^	1.23	0.73	< 0.0001^a,b^	0.46	< 0.0001^a,b^
AVN	MHD	0.38	0.58	< 0.0001^a,b^	0.43	0.0002^a,b^	0.72	0.83	< 0.0001^a,b^	0.93	< 0.0001^a,b^
	LA	1.48	0.59	< 0.0001^a,b^	0.30	0.0026^a,b^	1.5	0.59	0.0009^a,b^	0.85	< 0.0001^a,b^
	LV	0.3	0.54	< 0.0001^a,b^	0.30	0.0027^a,b^	1.57	0.90	< 0.0001^a,b^	0.97	< 0.0001^a,b^
	RA	1.77	0.81	< 0.0001^a,b^	0.77	< 0.0001^a,b^	0.48	0.65	0.0002^a,b^	0.63	< 0.0001^a,b^
	RV	0.36	0.57	< 0.0001^a,b^	0.38	0.0004^a,b^	0.55	0.79	< 0.0001^a,b^	0.90	< 0.0001^a,b^

*N/S*, node/structure.

a Significant results after Bonferroni correction.

b Sufficient statistical power in *post hoc* analysis.

### Comparison manual and autodelineation

The Dice similarity coefficient (DSC) for the SAN was 0.83 ± 0.103, and for the AVN, it was 0.75 ± 0.122. Both values exceed 0.7, indicating good spatial consistency between the automatic and manual contours (21).

The *D*_mean_ for manual-SAN and auto-SAN were 2.29 Gy (IQR, 0.89–4.16 Gy) and 2.2 Gy (IQR, 0.88–4.05 Gy), respectively. The *D*_mean_ for manual-AVN and auto-AVN was 2.7 Gy (IQR, 1.34–3.41 Gy) and 2.7 Gy (IQR, 1.41–3.34 Gy). The *D*_max_ for manual-SAN and auto-SAN were 3.56 Gy (IQR, 1.49–5.24 Gy) and 3.56 Gy (IQR, 2.22–5.10 Gy), respectively. The *D*_max_ for manual-AVN and auto-AVN were 3.20 Gy (IQR, 1.96–6.35 Gy) and 3.44 Gy (IQR, 2.20–6.25 Gy) (*p* > 0.05). The results indicated no significant differences in dosage between manual and automatic delineation. Additionally, there were no significant differences in *V*_1.0_, *V*_2.0_, and *V*_5.0_ between manual and automatic contouring (*p* > 0.05), indicating similar volume coverage at different dose levels (**Supplementary Figure S1**; **Table 3**).

**Table 3:** Delineation difference analysis of manual and automatic SAN and AVN.

Parameter	Node	SAN	*p*-value	AVN	*p*-value
		Median (IQR)		Median (IQR)	
*D*_mean_	Manual	2.29 (0.89–4.16)	0.72	2.7 (1.34–3.41)	0.96
	Auto	2.2 (0.88–4.05)		2.7 (1.41–3.34)	
*D*_max_	Manual	3.56 (1.49–5.24)	0.91	3.20 (1.96–6.35)	0.90
	Auto	3.56 (2.22–5.10)		3.44 (2.20–6.25)	
*V*_1.0_ (%)	Manual	100.00 (27.19–100)	0.92	100.00 (90.4–100)	0.87
	Auto	100.00 (19.19–100)		100.00 (93.48–100)	
*V*_2.0_ (%)	Manual	64.61 (0.00–100)	0.98	82.58 (0.00–100)	0.90
	Auto	52.65 (0.28–100)		68.39 (1.68–100)	
*V*_5.0_ (%)	Manual	0.00 (0.00–1.88)	0.94	0.00 (0.00–6.38)	0.70
	Auto	0.00 (0.00–0.87)		0.00 (0.00–4.73)	
Volume	Manual	4.48 (4.48–4.48)	0.03	4.48 (4.48–4.48)	0.03
	Auto	5.08 (4.17–5.42)		5.05 (3.84–6.35)	

*V_1.0_*, the volume of a structure receiving 1.0 Gy or more; *NA*, not applicable.

## Discussion

This study provides a relatively rare analysis of the radiotherapy dose to substructures of the cardiac conduction system in BC patients treated with IMRT. Furthermore, it examines the robustness and accuracy of a U-Net neural network deep learning model for autosegmentation of the SAN and AVN nodes.

Dosimetric analysis in this study reveals a critical finding: regardless of whether Three-dimensional conformal radiotherapy (3D-CRT) or IMRT is employed, the SAN in patients with right-sided breast cancer can become a relative dose hotspot. Specifically, with IMRT, the *D*_mean_ to the SAN reached 5.43 Gy, compared with an MHD of only 3.39 Gy. This finding aligns with earlier work by Errahmani et al. (22), in which 3D-CRT resulted in a SAN *D*_mean_ of 1.57 Gy and an MHD of 0.6 Gy. These data suggest that conventional MHD may underestimate the actual radiation exposure to the SAN, a critical cardiac substructure. Notably, IMRT significantly accentuates this discrepancy, increasing the SAN dose to approximately 3.5 times that observed with 3D-CRT.

The disparities in SAN dose levels observed between the two technologies are attributable to their fundamentally different dose distribution physics. 3D-CRT uses a limited tangent field with a steep dose gradient. High-dose areas are concentrated in anterior cardiac structures (e.g., the left anterior descending artery), whereas the SAN, located behind the edge of the irradiation field, receives a lower dose (5, 23). In contrast, IMRT significantly increases the volume of the heart receiving low-dose irradiation (e.g., V5 Gy) through multifield irradiation and intensity modulation, while achieving excellent target-area conformity (5). This widespread “low-dose bath” continuously exposes structures such as the SAN (located in the upper posterior part of the heart) to overlapping radiation, resulting in a significant increase in the dose they receive (7). This suggests that modern radiotherapy for right-sided breast cancer may shift cardiac risk from the traditional anterior wall ischemia model to a more accurate posterior electrophysiological structural damage model.

Yang et al. (24) reported that a 15-year follow-up of BC patients postradiotherapy showed cumulative incidence rates of arrhythmia, ischemic heart disease, and heart failure of 11.0%, 5.7%, and 4.8%, respectively. This underscores the importance of monitoring the relationship between radiation-induced damage and arrhythmia. Guha et al. (25) conducted a retrospective study of 85,423 adenocarcinoma patients aged 66 or older and found that the incidence of new atrial fibrillation in BC patients was 3.3% 1 year after treatment, double the rate observed in the nonbreast cancer group (1.8%). The observed *D*_mean_ level of 5.43 Gy for the SAN in this study is of clear clinical significance and warrants caution. Research conducted by Apte et al. (12) has established that a history of radiotherapy serves as an independent risk factor for the development of atrial fibrillation. Furthermore, van den Bogaard et al. (26) identified cardiac V5 Gy as a robust predictor of coronary events, thereby indirectly underscoring the pathological relevance of the low-to-moderate dose range. Notably, the study by Errahmani et al. (27) provides direct evidence of a positive correlation between the irradiation dose received by the RA and the risk of arrhythmia (OR = 1.19). It has been established that exposure to doses exceeding 5 Gy within the SAN, located in the RA wall, is sufficient to provoke localized inflammation and fibrosis. This modification of the electrophysiological microenvironment significantly increases the probability of subsequent sinus node dysfunction, bradycardia, and various atrial arrhythmias, including atrial fibrillation.

The RA profile is characterized by its clarity and ease of delineation in a standardized manner, making it a highly promising alternative indicator for assessing the risk to the cardiac conduction system. This study, in concordance with the existing literature (27), demonstrated a high degree of correlation between the SAN/AVN dose and the RA dose. Both RA *D*_mean_ and RA V5 Gy are shown to be effective screening tools, indicating an increased risk to the conduction system when elevated. The RA is a voluminous chamber, and its average dose does not accurately reflect the true irradiation of the SAN/AVN, particularly within the dose gradient region, where the substitution relationship may be significantly biased. Caution is therefore necessary when assessing RA doses as a surrogate. When feasible, it is recommended that the outlined SAN/AVN region be delineated in the planning system for direct evaluation, with reference to published anatomical atlases (17). Initially, a SAN *D*_mean_ of < 5 Gy should be used as one of the optimization goals, while strictly controlling the cardiac V5 Gy volume. This study confirms that the irradiated dose to the conduction system in right-sided breast cancer radiotherapy has become a new, MHD-masked risk concern in the IMRT era. We suggest that the conventional notion of negligible cardiac risk in right-sided breast cancer should be abandoned in favor of refined substructural dose assessment. The *D*_mean_ of the SAN and AVN should be actively constrained to < 5 Gy, and the volume receiving low-to-intermediate doses (e.g., V5 Gy) must be minimized. This approach is particularly critical for high-risk patients, such as those with pre-existing conduction abnormalities or younger individuals.

To support accurate dose assessment, this study developed and validated a deep learning model for automatic segmentation of key substructures of the cardiac conduction system—the SAN and AVN—on CT images from radiotherapy plans. The model was based on a two-dimensional U-Net architecture and demonstrated commendable geometric accuracy (SAN DSC: 0.83 ± 0.103; AVN DSC: 0.75 ± 0.122). Furthermore, no statistically significant differences were observed in key radiotherapy dosimetric parameters between automated and expert manual outlining, providing a reliable tool for subsequent accurate dose assessment and risk studies. The model employs the classical 2D U-Net architecture, which has been extensively validated for its efficacy in biomedical image segmentation, efficient training characteristics, and adaptability to medium-sized datasets (13). The U-Net architecture, featuring an encoder–decoder design with skip connections, has been shown to effectively integrate local image details with global semantic contextual information (13). This is critical for localizing and segmenting small targets that are highly dependent on fixed anatomical adjacencies (e.g., the superior vena cava–right atrial junction area, AVN vs. Koch’s triangle) in low-contrast CT images. The results demonstrate that even with a relatively basic 2D architecture, the deep learning model can autonomously learn these highly specific anatomical features in a data-driven manner.

The present work represents a significant advancement compared to previous studies. For example, Loap et al. (17, 18) provided a viable solution for dose assessment of these critical structures by integrating manually mapped SAN and AVN atlases into automated atlas-based segmentation (ABAS) software, but their reported segmentation accuracy was limited (Dice similarity coefficients: 0.56 for SAN and 0.15 for AVN). In contrast, the deep learning-based approach used in this study achieves a significant improvement in segmentation performance, with Dice coefficients of 0.83 and 0.75 for SAN and AVN, respectively. These results highlight the advantages of data-driven deep learning methods over traditional atlas-alignment techniques that rely on anatomical templates.

To address the challenge of limited medical image data and to enhance the model’s generalization ability, this study implements a rigorous data augmentation and regularization strategy designed to simulate possible anatomical variations and scanning differences in clinical images, providing an effective approach to improving model robustness (14). Furthermore, an early stopping strategy based on validation set performance ensures effective convergence during training. The training and validation loss curves decrease synchronously without significant divergence, which intuitively reflects the model’s stable learning process and strong generalization potential.

The most critical clinical finding of this study is that there is no significant difference between automated and manual outlining in the dosimetric parameters that determine clinical risk. This directly addresses the fundamental question of whether automated segmentation tools can be effectively used for clinical dose assessment and plan optimization. This finding aligns with the results of Shen et al. and Van der Vorst et al., who state that dosimetric validation is the “gold standard” for evaluating the clinical suitability of automated segmentation (4, 28). Consequently, our model represents a robust tool that can be directly applied in large-scale retrospective cohort studies to investigate the relationship between cardiac conduction system dose exposure and the risk of arrhythmias.

This study has several limitations. Its retrospective, single−center design and limited sample size may constrain generalizability, although a *post hoc* power analysis supported the reliability of the primary findings. Dose assessments were derived from free−breathing CT without cardiac motion correction, which could introduce uncertainty. Moreover, the conclusion of dosimetric equivalence has not been correlated with clinical arrhythmia endpoints and requires prospective validation. Finally, the applicability of our results to other radiotherapy techniques remains to be investigated.

Future multicenter prospective studies with larger cohorts are warranted to validate the robustness and generalizability of the model. Applying the model in such settings will enable the establishment of dose–response relationships between SAN/AVN irradiation and clinical arrhythmic endpoints, thereby providing direct evidence to guide the development of individualized cardiac substructure dose constraints for more precise cardioprotective radiotherapy.

## Conclusions

During breast IMRT for right-sided BC patients, the SAN is significantly exposed, and the RA can serve as a reliable surrogate for predicting SAN and AVN doses. Deep learning-based autosegmentation enables robust delineation of these nodes, supporting accurate dosimetry assessment.

## Data Availability

The original contributions presented in the study are included in the article/**Supplementary Material**. Further inquiries can be directed to the corresponding author.
