# Supplementary material for: Radiation exposure and clinical validation of autosegmentation models for the supraventricular cardiac conduction system in breast cancer radiotherapy: an institutional perspective
Source: Front Oncol. 2026 Jan 29;16:1734696. doi: 10.3389/fonc.2026.1734696 (PMC12893944; doi:10.3389/fonc.2026.1734696)
Supplement: Supplementary file 1 [file DataSheet1.docx]

**Deep Learning Methodology for Auto-segmentation of SAN and AVN in Non-Contrast CT**

We included a total of 65 patients who underwent non-contrast planning computed tomography (CT) with corresponding manually delineated contours of the sinoatrial node (SAN) and atrioventricular node (AVN). To ensure methodological rigor and minimize overfitting, the dataset was randomly partitioned into three independent subsets，training set (60 patients, 420 Axial Slices） for model parameter optimization, validation set （7 patients, 49 Axial Slices) for hyperparameter tuning, training monitoring, and early stopping, Test set （20 patients, 140 Axial Slices) for Independent final performance evaluation.

We employed a two-dimensional U-Net architecture as the core segmentation network. The model was implemented using PyTorch 2.0.0 within a Python 3.8.10 environment. All training and inference procedures were accelerated using an NVIDIA TITAN RTX GPU with 24 GB of memory. To ensure robust evaluation and mitigate overfitting, the dataset was randomly partitioned into three subsets: Training set (60 patients），validation set (7 patients）, and Test set (20 patients）. The training parameters are: (1) Mode: unet_2d; (2) batch size: 6; (3) Learning Rate: 1e-4; (4) loss function: Binary Cross Entropy loss; (5) Max Epochs: 100. The optimizer uses Adam.

Data augmentation strategy was used to improve the generalization ability of the model. Specifically, augmentation operations were performed on each training sample with 40% probability, including randomly selecting a fixed rotation angle from 0°/90°/180°/270° and adding additional micro-rotation perturbations ranging from −10° to +10°; random clipping was performed based on random offset (0-20 pixels). The horizontal flip and vertical flip were performed with 50% probability. ColorJitter (brightness ±0.2, contrast ±0.2, hue ±0.02) was applied. An early stop strategy based on the performance of the validation set was used to ensure that the training process was transparent and reproducible, and overfitting was effectively avoided. The training/validation loss curve is as follows:

**
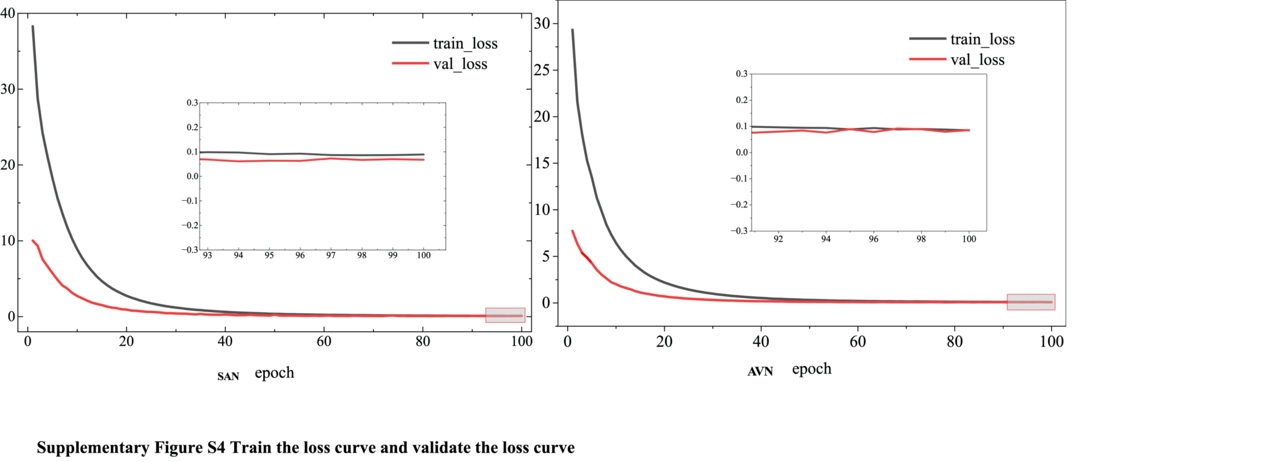
**

Both the training loss curve and the validation loss curve decreased rapidly with the training rounds and reached a plateau together after about the 50th round. Importantly, the two curves were always closely accompanied throughout the training process and no obvious bifurcation was observed. This indicates that the learning process of our model is stable and converges well, and there is no significant overfitting phenomenon, which indirectly supports that the model has good generalization ability.

**Auto‑segmentation training details**

**Provide more details on training and validation: exact number of training/validation/test slices or volumes, data augmentation strategies, cross‑validation (if any), stopping criteria, and how hyperparameters were selected. Report training/validation loss curves and any measures taken to avoid overfitting.**

We thank the reviewer for this request for greater methodological transparency. The AVN data consisted of 60 training cases (420 slices), 7 validation cases (49 slices), and 20 test cases (140 slices). The SAN data consisted of 60 training cases (420 slices), 7 validation cases (49 slices), and 20 test cases (140 slices). Data augmentation strategy was used to improve the generalization ability of the model. Specifically, augmentation operations were performed on each training sample with 40% probability, including randomly selecting a fixed rotation Angle from 0°/90°/180°/270° and adding additional micro-rotation perturbations ranging from −10° to +10°; Random clipping was performed based on random offset (0-20 pixels). The horizontal flip and vertical flip were performed with 50% probability. Color Jitter (brightness ±0.2, contrast ±0.2, hue ±0.02) was applied. An early stop strategy based on the performance of the validation set was used to ensure that the training process was transparent and reproducible, and overfitting was effectively avoided. The training/validation loss curves are shown in Supplementary Figure 4.
