# Supplementary material for: Radiation exposure and clinical validation of autosegmentation models for the supraventricular cardiac conduction system in breast cancer radiotherapy: an institutional perspective
Source: Front Oncol. 2026 Jan 29;16:1734696. doi: 10.3389/fonc.2026.1734696 (PMC12893944; doi:10.3389/fonc.2026.1734696)
Supplement: Supplementary file 2 [file DataSheet2.docx]

**post hoc power analysis**

Supplementary Table 1 **post hoc power analysis of left-side BC**

| Node | structure |  | Left-side BC | | | | | |  |
| --- | --- | --- | --- | --- | --- | --- | --- | --- | --- |
|  |  | Ratio | | Correlation | |  | Liner regression | |  |
|  |  | N/ S | | *r* | *P* value | ***Power (1-β)*** | *R^2^* | *P* value | ***Power (1-β)*** |
| SAN | MHD | 0.19 | | 0.36 | 0.005 | **60%** | 0.13 | 0.0052 | **61.9%** |
|  | LA | 0.68 | | 0.53 | <0.0001 | **96.8%** | 0.18 | 0.0009 | **82.2%** |
|  | LV | 0.14 | | 0.22 | 0.099 | **18.3%** | 0.05 | 0.078 | **18.2%** |
|  | RA | 0.82 | | 0.80 | <0.0001 | **>99.9%** | 0.63 | <0.0001 | **>99.9%** |
|  | RV | 0.17 | | 0.44 | 0.0005 | **83.6%** | 0.15 | 0.002 | **71%** |
| AVN | MHD | 0.38 | | 0.58 | <0.0001 | **99.2%** | 0.43 | 0.0002 | **>99.9%** |
|  | LA | 1.48 | | 0.59 | <0.0001 | **99.4%** | 0.30 | 0.0026 | **98.9%** |
|  | LV | 0.3 | | 0.54 | <0.0001 | **97.5%** | 0.30 | 0.0027 | **98.9%** |
|  | RA | 1.77 | | 0.81 | <0.0001 | **>99.9%** | 0.77 | <0.0001 | **>99.9%** |
|  | RV | 0.36 | | 0.57 | <0.0001 | **98.9%** | 0.38 | 0.0004 | **>99.9%** |

**Supplementary Table 2 post hoc power analysis of right-side BC**

| Node | structure |  | Right-side BC | | | | | |  |
| --- | --- | --- | --- | --- | --- | --- | --- | --- | --- |
|  |  | Ratio | | Correlation | |  | Liner regression | |  |
|  |  | N/ S | | *r* | *P* value | ***Power (1-β)*** | *R^2^* | *P* value | ***Power (1-β)*** |
| SAN | MHD | 1.89 | | 0.77 | <0.0001 | **99.5%** | 0.47 | <0.0001 | **98.1%** |
|  | LA | 2.89 | | 0.58 | 0.0012 | **80%** | 0.59 | <0.0001 | **>99.9%** |
|  | LV | 3.24 | | 0.61 | 0.001 | **84.4%** | 0.25 | <0.0001 | **61.1%** |
|  | RA | 1.11 | | 0.93 | <0.0001 | **>99.9%** | 0.63 | <0.0001 | **>99.9%** |
|  | RV | 1.23 | | 0.73 | <0.0001 | **98.3%** | 0.46 | <0.0001 | **97.7%** |
| AVN | MHD | 0.72 | | 0.83 | <0.0001 | **>99.9%** | 0.93 | <0.0001 | **>99.9%** |
|  | LA | 1.5 | | 0.59 | 0.0009 | **80.2%** | 0.85 | <0.0001 | **>99.9%** |
|  | LV | 1.57 | | 0.90 | <0.0001 | **>99.9%** | 0.97 | <0.0001 | **>99.9%** |
|  | RA | 0.48 | | 0.65 | 0.0002 | **91.1%** | 0.63 | <0.0001 | **>99.9%** |
|  | RV | 0.55 | | 0.79 | <0.0001 | **99.7%** | 0.90 | <0.0001 | **>99.9%** |

**Correlation analysis post hoc power calculation steps**

1. Select the test type
   1. Open the G*Power software
   2. Select Exact in Test family

1.3 In the Statistical test, select Correlation: Bivariate normal model. The data in this study are non-normally distributed, but because there is no non-normally distributed model for gpower, the approximation can be calculated by Correlation: Bivariate normal model

2. Enter the parameters

Prepare and enter the following parameters for each correlation analysis of (for each row in the table):

2.1 The Tail(s): choose Two (s)

2.2 Alpha err prob (alpha error probability): significance level, this study was corrected for multiple comparisons, using Bonferroni more positive α=0.01

2.3 Total sample size (N): 59 for left analysis and 28 for right analysis

2.4 Correlation ρ H1 (alternative hypothesis correlation ρ): In post hoc analysis, this should be the correlation coefficient r calculated from the study

3. Calculate and read power

3.1 Ensure that the Correlation ρ H0 (the null hypothesis correlation ρ) is 0

3.2 Click the Calculate button in the upper right corner

3.3 The value shown in Power (1-β err prob) in the result box is the statistical power

**linear regression was used to analyze post hoc power calculation steps**

1. Select the test type

1.1 Open the G*Power software

1.2 Select F tests in Test family

1.3 In the Statistical test, select: Linear multiple regression: Fixed model, R² deviation from zero.

2. Enter the parameters

2.1 Effect size f²: This is the most critical parameter. It's calculated by the R squared value. α err prob (alpha error probability): significance level. It is usually set to 0.05. In this study, Bonferroni was used to compare the positive α=0.01.

2.2 Total sample size (N): 59 for left analysis and 28 for right analysis.

2.3 Number of predictors: the total number of independent variables in the input model. Simple linear regression, this number is 1.

3. Calculate the effect size f²

3.1 Click the Determine => button next to the Effect size f² input box.

3.2 A new window will appear. In the Squared multiple correlation ρ² field, the R² value calculated from the study is entered.

3.3 Click the Calculate button.

3.4 The software will automatically fill in the calculated value in the Effect size f² field in the main window.

3.5 Click Calculate and transfer to main window to send the effect size back to the main window.

4. Calculate and read power

4.1 Return to the main window and click the Calculate button.

4.2 The value shown in Power (1-β err prob) in the result box is the statistical power.

**Residual analysis of linear correlation regression analysis between SAN and RA dose in left-sided breast cancer**


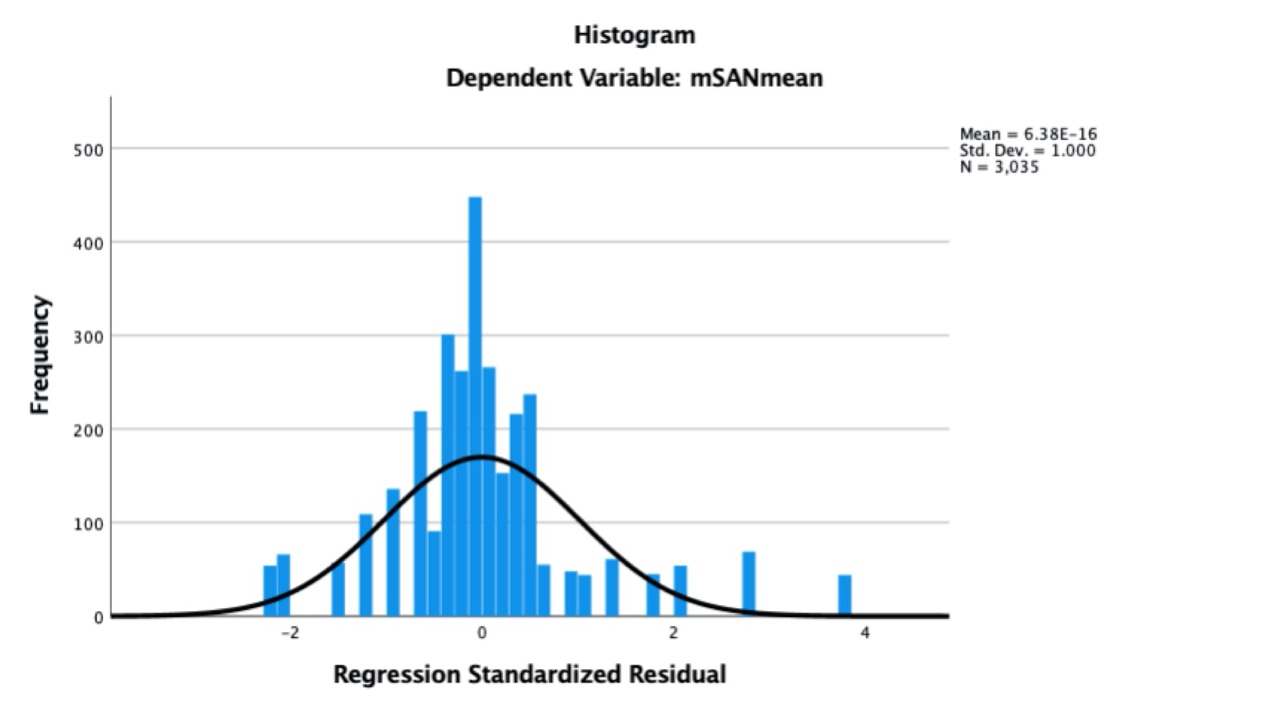


Supplementary Figure 5. Histogram of regression Standardized residuals

The normality of the residuals was judged and showed a normal distribution


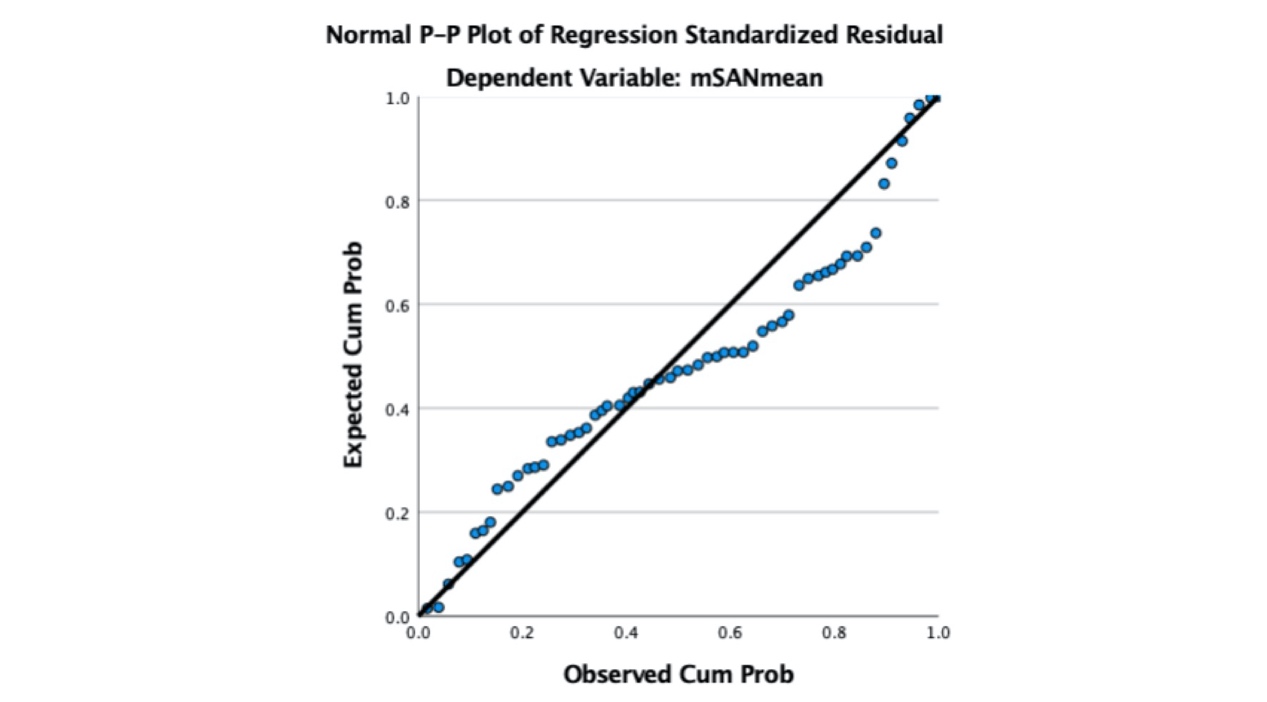


Supplementary Figure 6. Normal P-P plot of regression standardized residuals

It is suggested that the residuals follow approximately a normal distribution


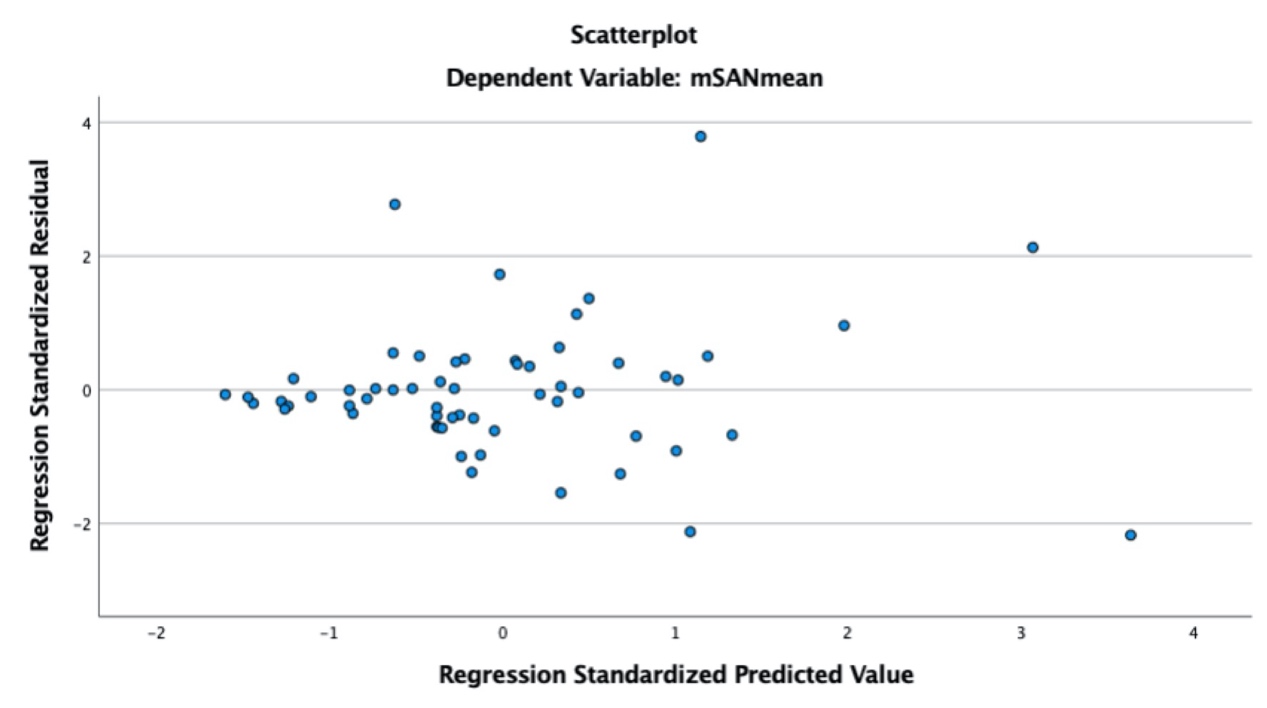


Supplementary Figure 7. Scatter Plot of Standardized Residuals

The predicted value and the corresponding residual were distributed uniformly, and no special distribution form appeared, suggesting that the variance of the residual was homogeneous


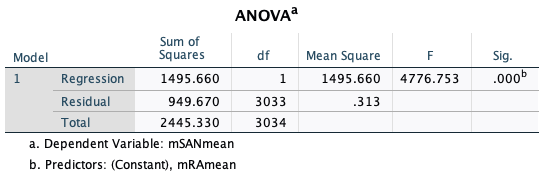


Supplementary Figure 8. Model Statistical test results, using ANOVA, showed that the regression model was statistically significant

**Residual analysis of linear correlation regression analysis between SAN and RA dose in right breast cancer**


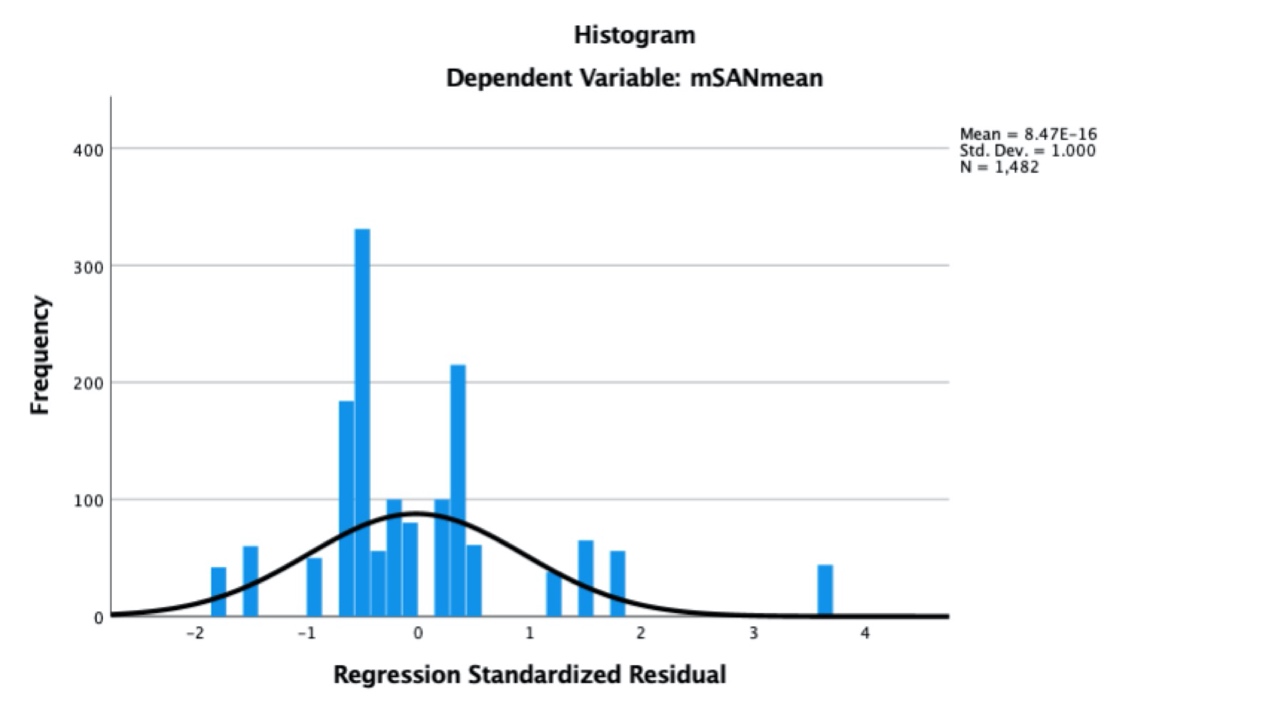


Supplementary Figure 9. Histogram of regression standardized residuals

The normality of the residuals was judged and showed a normal distribution


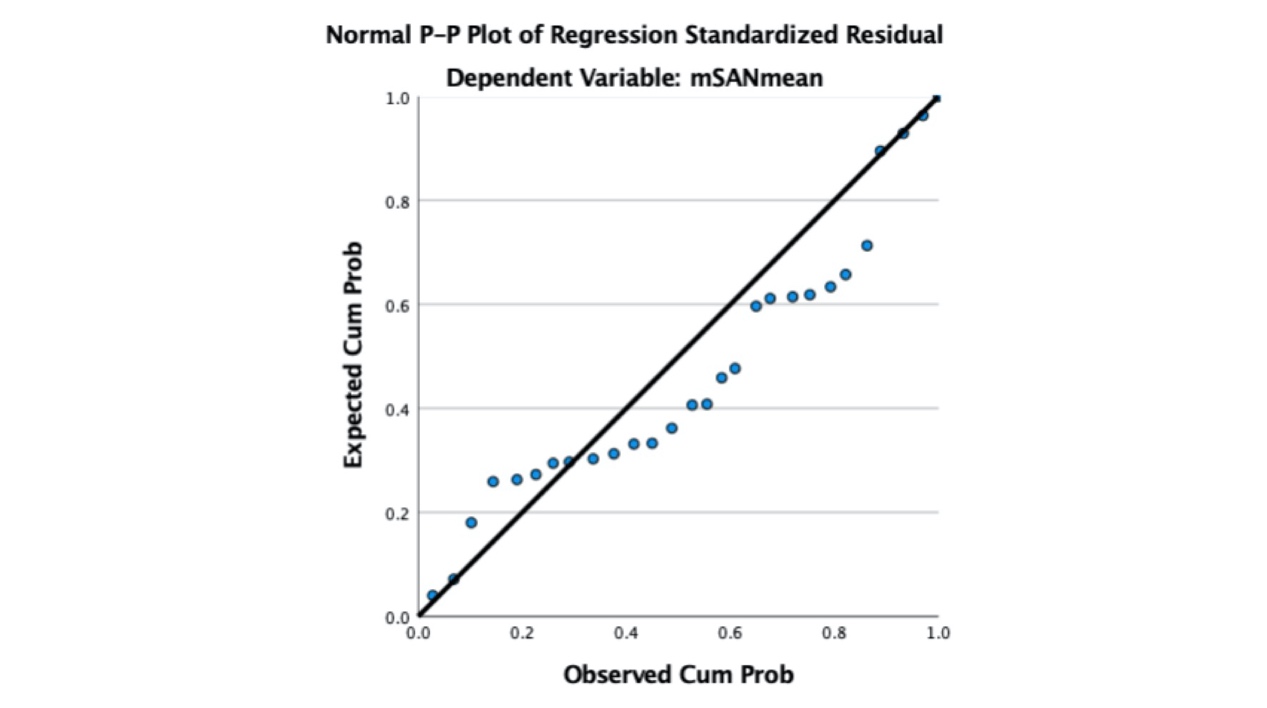


Supplementary Figure 10. Normal P-P plot of regression standardized residuals

It is suggested that the residuals follow approximately a normal distribution


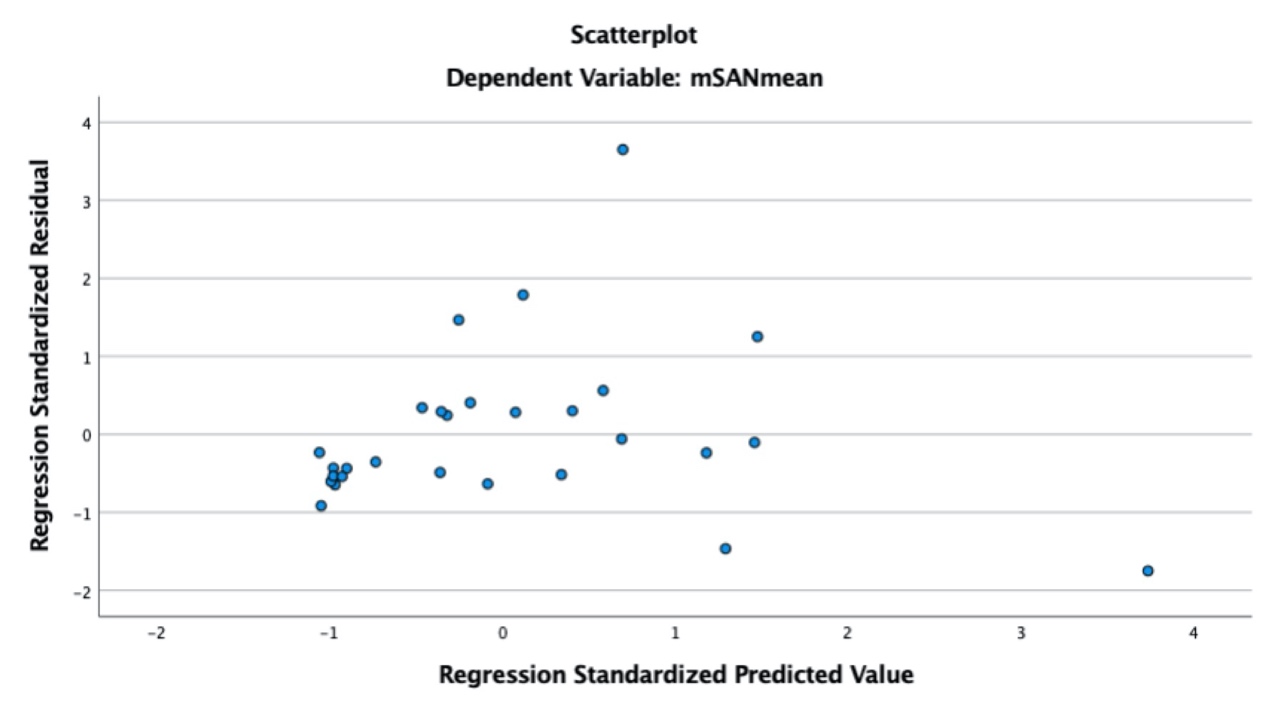


Supplementary Figure 11. Scatter Plot of Standardized Residuals

The predicted value and the corresponding residual were distributed uniformly, and no special distribution form appeared, suggesting that the variance of the residual was homogeneous


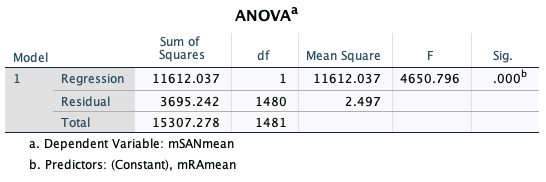


Supplementary Figure 12. Model Statistical test results, using ANOVA, showed that the regression model was statistically significant
