# Supplementary material for: Radiation exposure and clinical validation of autosegmentation models for the supraventricular cardiac conduction system in breast cancer radiotherapy: an institutional perspective
Source: Front Oncol. 2026 Jan 29;16:1734696. doi: 10.3389/fonc.2026.1734696 (PMC12893944; doi:10.3389/fonc.2026.1734696)
Supplement: Supplementary file 3 [file DataSheet3.docx]

**
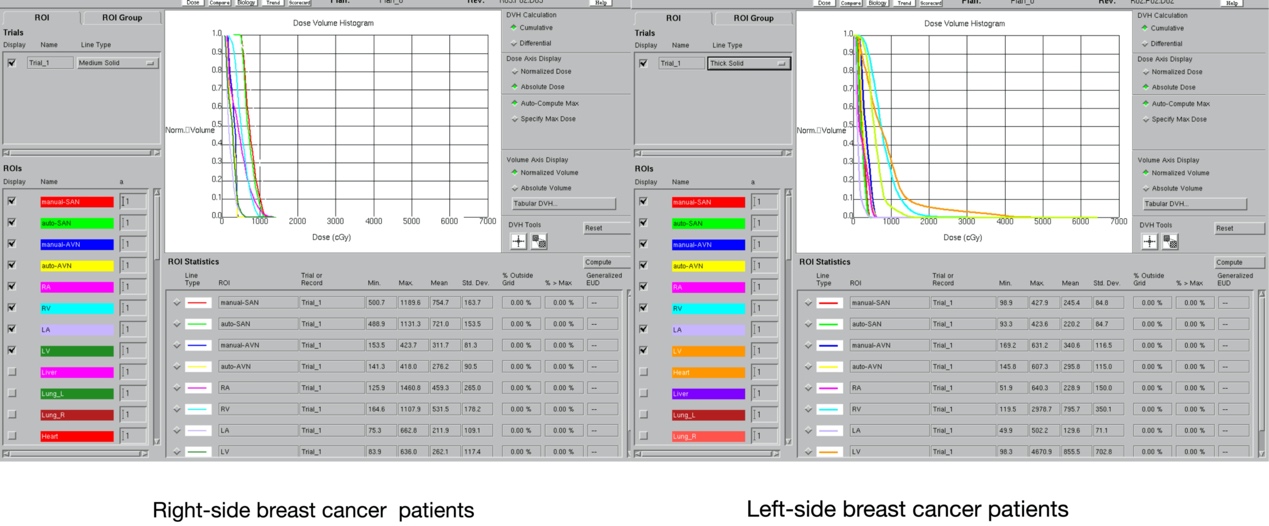
**

**Supplementary Figure1 DVH for manual and auto segmentation of SAN and AVN**

**Supplementary_Material_1_Cardiac_Contouring**

**Manual Delineation and Multi-disciplinary Review Process**

Given the inherent challenges in delineating low-contrast cardiac substructures on non-contrast planning CT scans, a rigorous multi-disciplinary review workflow was implemented to ensure contouring accuracy and clinical relevance.

All contours were initially delineated manually by an attending radiation oncologist using a standardized cardiac atlas [1-3] as the primary reference. To optimize visualization of relevant tissues, all CT images (acquired at 3 mm slice thickness) were reviewed with a consistent window width of 400 Hounsfield Units (HU) and a level of 40 HU. This setting was critical for distinguishing the cardiac chambers (approximately 40-50 HU), myocardium (30-50 HU), and the adjacent pericardial fat pad (-100 to -50 HU).

Key immutable or relatively fixed anatomical landmarks—including bony structures, cartilages, the roots of the great vessels, and fat planes (e.g., the atrioventricular groove fat and pericardial fat pad)—were utilized to compensate for the lack of soft-tissue contrast and to verify anatomical positioning.

Following the initial contouring, a mandatory two-step peer-review process was conducted:

1. **Anatomical Accuracy Review**: All contours were reviewed by a chief diagnostic radiologist, who assessed the anatomical plausibility. This evaluation focused on the correct identification of septal structures, the appropriate inclusion or exclusion of atrial appendages, and the accurate utilization of fat planes as boundaries.
2. **Clinical Dosimetric Review**: Subsequently, a senior radiation oncologist reviewed the contours for clinical and dosimetric utility, ensuring they were suitable for dose-volume analysis and treatment plan optimization, and that their size did not erroneously influence planning decisions.

Any discrepancies identified between the reviewers were resolved through a consensus discussion with reference to the standard delineation atlas, and contours were modified accordingly before final approval.

| substructure | Core anatomical landmarks and delineation methods | Upper bound/lower bound | Key tips and considerations |
| --- | --- | --- | --- |
| Heart | Outer margin: The inner margin of pericardial fat  Apex: The very tip of the heart muscle  At the bottom of the heart: at the bifurcation level of the pulmonary artery trunk | Upper boundary: The lower edge of the bifurcation of the pulmonary artery trunk  Lower boundary: The inferior edge of the apex of the left ventricle | It includes all the heart chambers, the roots of blood vessels and the pericardium |
| Left ventricle/right ventricle | Boundary: Ventricular septum, the soft tissue septum between the left and right ventricles.  Boundary of the cardiac chamber: endocardial surface  Atrioventricular groove: The fatty space between the ventricles and the atria | Upper boundary: aortic/pulmonary valve inferior level  Lower boundary: apex of heart | The contour of the ventricular septum was ensured to be intact and to avoid overlapping left and right ventricular contours |
| Left atrium/right atrium | The atrial septum, which can be divided along a virtual line from the midpoint of the posterior edge of the aortic root to the midpoint of the posterior wall of the heart  Atrioventricular groove: boundary with the ventricle | Upper boundary: the level of the entrance of the superior vena cava (right atrium)  Lower boundary: level of atrioventricular sulcus | The left atrium is last and in front is the aorta  The right atrium is rightmost, and the septum is medial to it |
| SAN | Unable to anatomically delineate, a sphere 2cm in diameter was generated anterolateral to the junction of the superior vena cava and the right atrium, according to the method of Loap et al | The center of the sphere was usually located on the axial radiograph at the level of the ascending segment of the aortic root | This is a geometric surrogate volume designed to cover the anatomical region containing the SAN and its motion uncertainties |
| **AVN** | Unable to anatomically delineate, alternative methods typically simplify to generate a sphere 2cm in diameter at the lower part of the atrial septum, near the center of the heart, in the Koch triangle region bounded by the ostium of the coronary sinus, tricuspid septal valve, and Todaro's tendon | The center of the sphere was often located approximately 1cm below the level where the left atrium first appeared, at the intersection of the heart | The same geometric surrogate volume was designed to cover the anatomical region containing the AVN and its motion uncertainties |

**Supplementary Figure 1. Schematic representation of the overall outline of the heart**

**
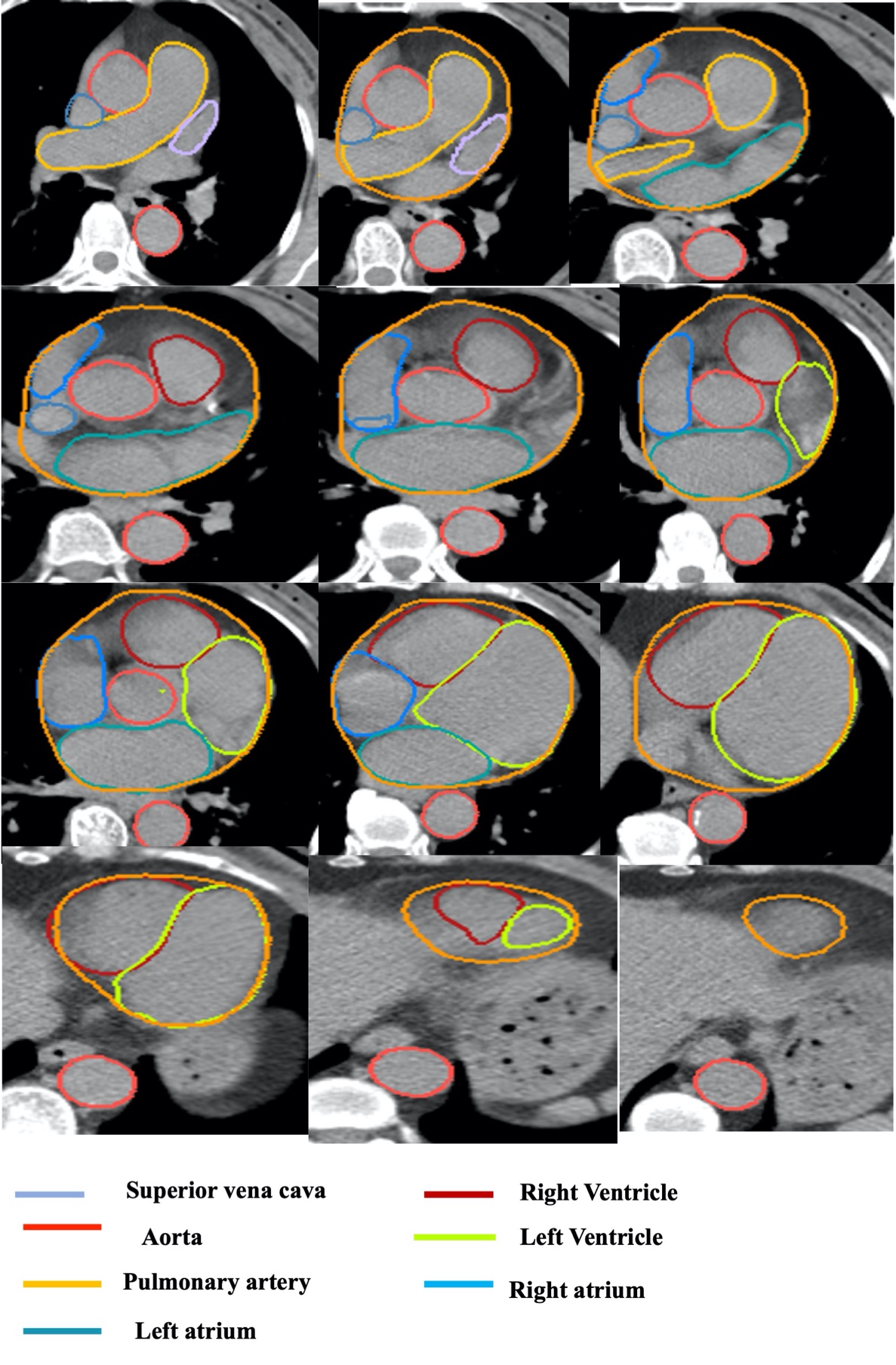
**

**Supplementary Figure 2. Steps for generating alternative structures of the SAN**


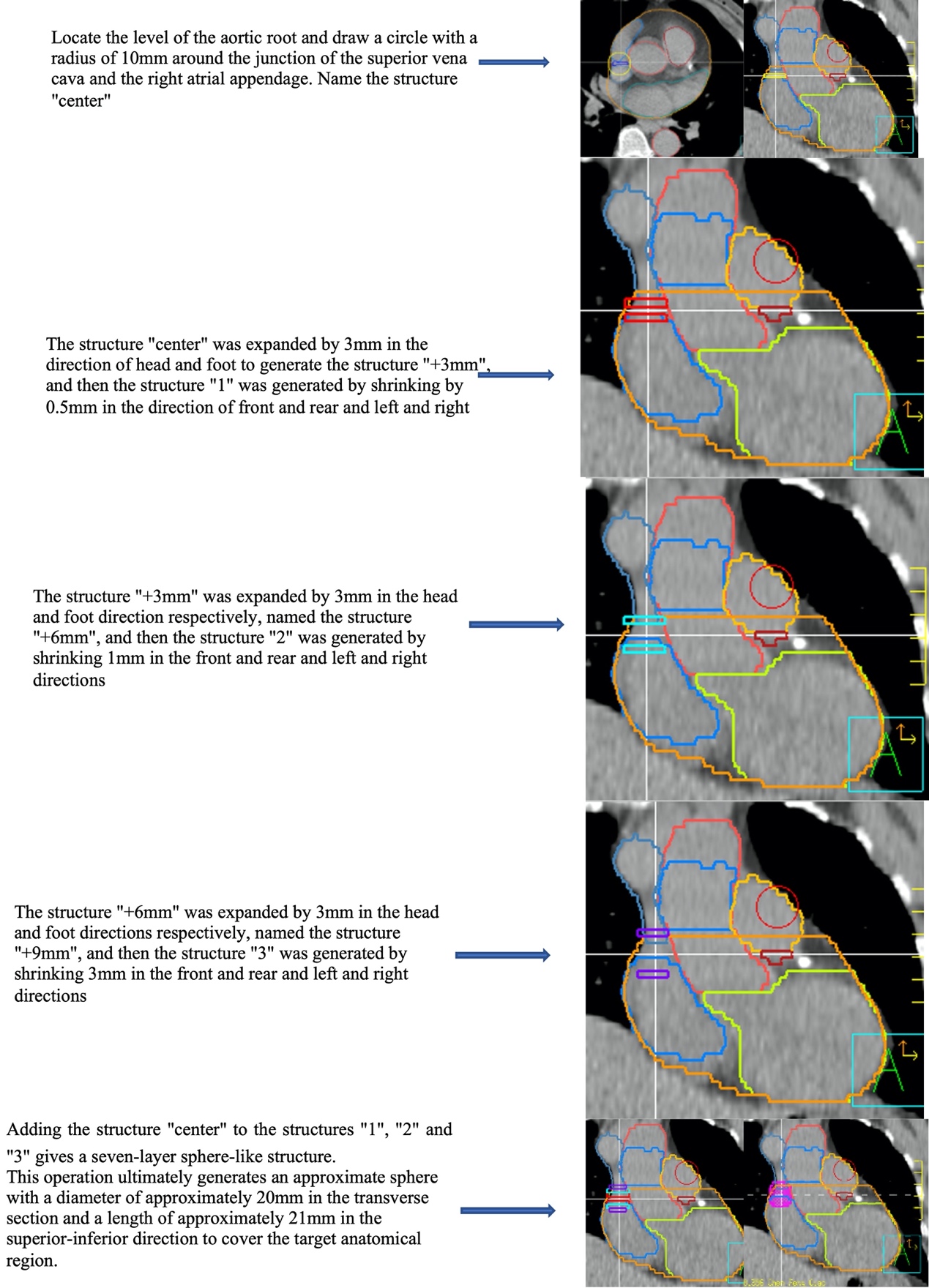


**Supplementary Figure 3. Steps for generating the AVN alternative structure**


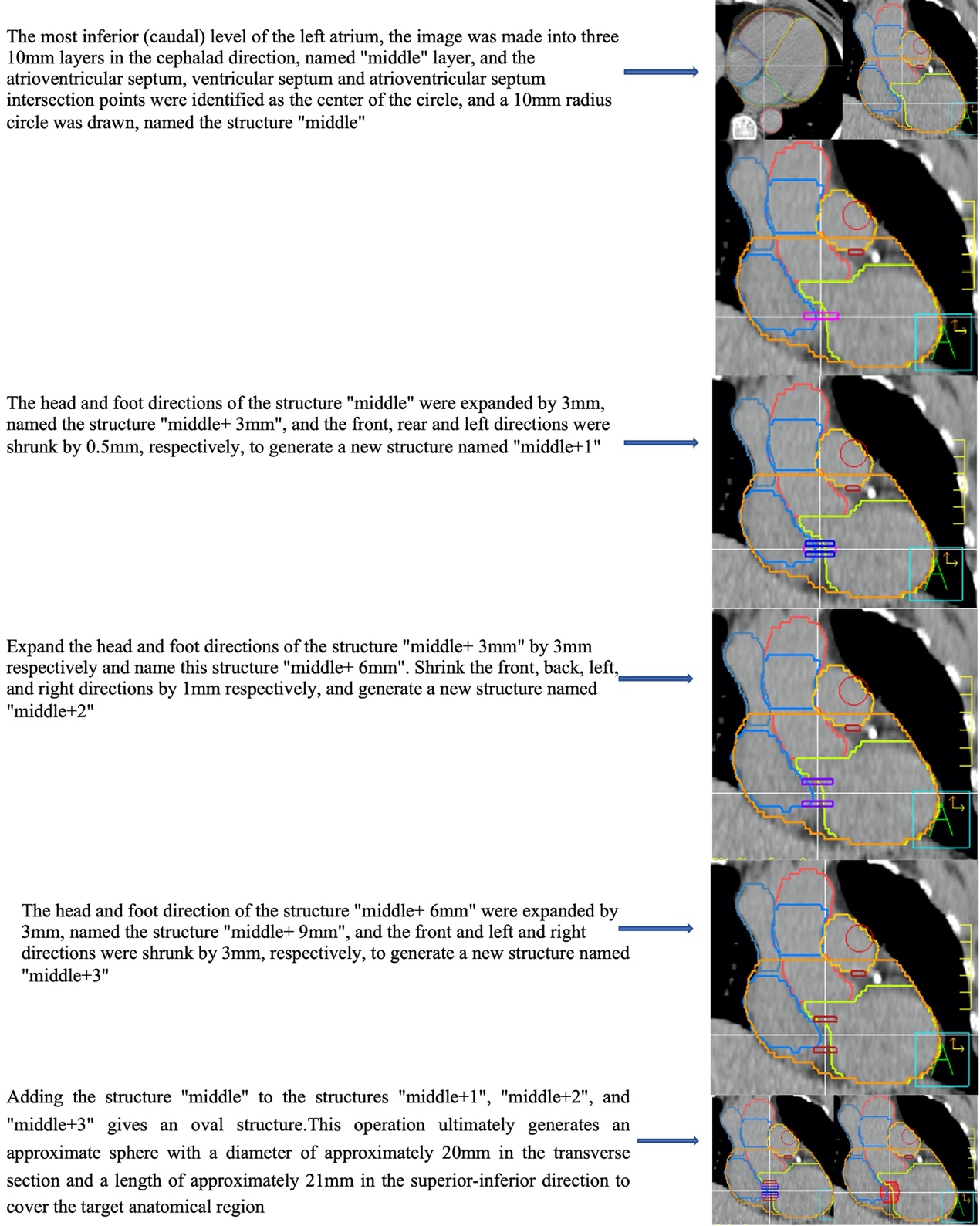


**References**

1. Feng M, Moran JM, Koelling T, Chughtai A, Chan JL, Freedman L, Hayman JA, Jagsi R, Jolly S, Larouere J *et al*: **Development and validation of a heart atlas to study cardiac exposure to radiation following treatment for breast cancer**. *Int J Radiat Oncol Biol Phys* 2011, **79**(1):10-18.

2. Milo MLH, Offersen BV, Bechmann T, Diederichsen ACP, Hansen CR, Holtved E, Josipovic M, Lörincz T, Maraldo MV, Nielsen MH *et al*: **Delineation of whole heart and substructures in thoracic radiation therapy: National guidelines and contouring atlas by the Danish Multidisciplinary Cancer Groups**. *Radiother Oncol* 2020, **150**:121-127.

3. Loap P, De Marzi L, Kirov K, Servois V, Fourquet A, Khoubeyb A, Kirova Y: **Development of Simplified Auto-Segmentable Functional Cardiac Atlas**. *Pract Radiat Oncol* 2022, **12**(6):533-538.

**Supplementary_Materials_2_autosegmention**

**Deep Learning Methodology for Auto-segmentation of SAN and AVN in Non-Contrast CT**

We included a total of 65 patients who underwent non-contrast planning computed tomography (CT) with corresponding manually delineated contours of the sinoatrial node (SAN) and atrioventricular node (AVN). To ensure methodological rigor and minimize overfitting, the dataset was randomly partitioned into three independent subsets，training set (60 patients, 420 Axial Slices） for model parameter optimization, validation set （7 patients, 49 Axial Slices) for hyperparameter tuning, training monitoring, and early stopping, Test set （20 patients, 140 Axial Slices) for Independent final performance evaluation.

We employed a two-dimensional U-Net architecture as the core segmentation network. The model was implemented using PyTorch 2.0.0 within a Python 3.8.10 environment. All training and inference procedures were accelerated using an NVIDIA TITAN RTX GPU with 24 GB of memory. To ensure robust evaluation and mitigate overfitting, the dataset was randomly partitioned into three subsets: Training set (60 patients），validation set (7 patients）, and Test set (20 patients）. The training parameters are: (1) Mode: unet_2d; (2) batch size: 6; (3) Learning Rate: 1e-4; (4) loss function: Binary Cross Entropy loss; (5) Max Epochs: 100. The optimizer uses Adam.

Data augmentation strategy was used to improve the generalization ability of the model. Specifically, augmentation operations were performed on each training sample with 40% probability, including randomly selecting a fixed rotation angle from 0°/90°/180°/270° and adding additional micro-rotation perturbations ranging from −10° to +10°; random clipping was performed based on random offset (0-20 pixels). The horizontal flip and vertical flip were performed with 50% probability. ColorJitter (brightness ±0.2, contrast ±0.2, hue ±0.02) was applied. An early stop strategy based on the performance of the validation set was used to ensure that the training process was transparent and reproducible, and overfitting was effectively avoided. The training/validation loss curve is as follows:

**
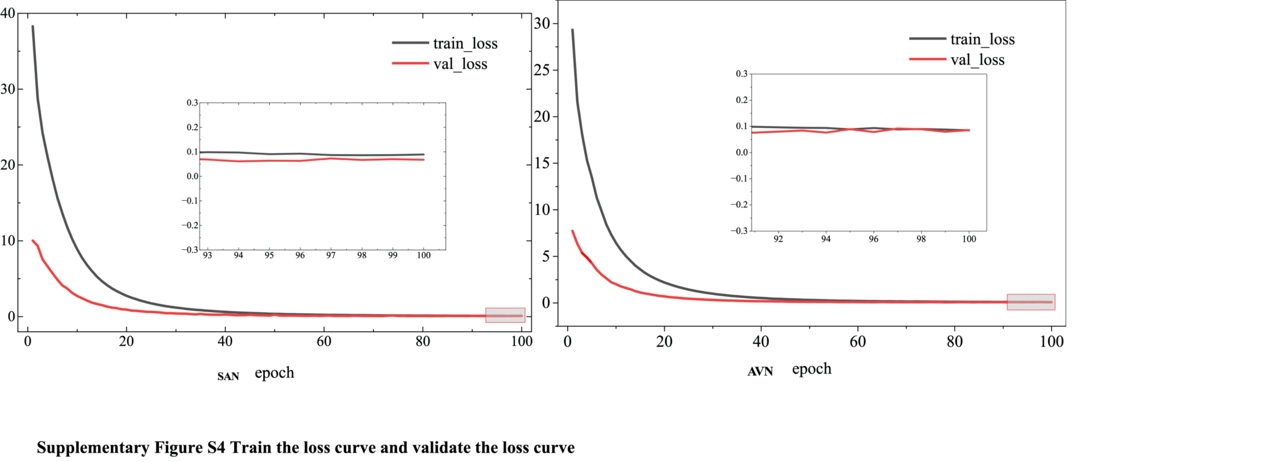
**

Both the training loss curve and the validation loss curve decreased rapidly with the training rounds and reached a plateau together after about the 50th round. Importantly, the two curves were always closely accompanied throughout the training process and no obvious bifurcation was observed. This indicates that the learning process of our model is stable and converges well, and there is no significant overfitting phenomenon, which indirectly supports that the model has good generalization ability.

**Auto‑segmentation training details**

**Provide more details on training and validation: exact number of training/validation/test slices or volumes, data augmentation strategies, cross‑validation (if any), stopping criteria, and how hyperparameters were selected. Report training/validation loss curves and any measures taken to avoid overfitting.**

We thank the reviewer for this request for greater methodological transparency. The AVN data consisted of 60 training cases (420 slices), 7 validation cases (49 slices), and 20 test cases (140 slices). The SAN data consisted of 60 training cases (420 slices), 7 validation cases (49 slices), and 20 test cases (140 slices). Data augmentation strategy was used to improve the generalization ability of the model. Specifically, augmentation operations were performed on each training sample with 40% probability, including randomly selecting a fixed rotation Angle from 0°/90°/180°/270° and adding additional micro-rotation perturbations ranging from −10° to +10°; Random clipping was performed based on random offset (0-20 pixels). The horizontal flip and vertical flip were performed with 50% probability. Color Jitter (brightness ±0.2, contrast ±0.2, hue ±0.02) was applied. An early stop strategy based on the performance of the validation set was used to ensure that the training process was transparent and reproducible, and overfitting was effectively avoided. The training/validation loss curves are shown in Supplementary Figure 4.

**Supplementary_Materials_3_Model residual analysis and post hoc power analysis**

**post hoc power analysis**

Supplementary Table 1 **post hoc power analysis of left-side BC**

| Node | structure |  | Left-side BC | | | | | |  |
| --- | --- | --- | --- | --- | --- | --- | --- | --- | --- |
|  |  | Ratio | | Correlation | |  | Liner regression | |  |
|  |  | N/ S | | *r* | *P* value | ***Power (1-β)*** | *R^2^* | *P* value | ***Power (1-β)*** |
| SAN | MHD | 0.19 | | 0.36 | 0.005 | **60%** | 0.13 | 0.0052 | **61.9%** |
|  | LA | 0.68 | | 0.53 | <0.0001 | **96.8%** | 0.18 | 0.0009 | **82.2%** |
|  | LV | 0.14 | | 0.22 | 0.099 | **18.3%** | 0.05 | 0.078 | **18.2%** |
|  | RA | 0.82 | | 0.80 | <0.0001 | **>99.9%** | 0.63 | <0.0001 | **>99.9%** |
|  | RV | 0.17 | | 0.44 | 0.0005 | **83.6%** | 0.15 | 0.002 | **71%** |
| AVN | MHD | 0.38 | | 0.58 | <0.0001 | **99.2%** | 0.43 | 0.0002 | **>99.9%** |
|  | LA | 1.48 | | 0.59 | <0.0001 | **99.4%** | 0.30 | 0.0026 | **98.9%** |
|  | LV | 0.3 | | 0.54 | <0.0001 | **97.5%** | 0.30 | 0.0027 | **98.9%** |
|  | RA | 1.77 | | 0.81 | <0.0001 | **>99.9%** | 0.77 | <0.0001 | **>99.9%** |
|  | RV | 0.36 | | 0.57 | <0.0001 | **98.9%** | 0.38 | 0.0004 | **>99.9%** |

**Supplementary Table 2 post hoc power analysis of right-side BC**

| Node | structure |  | Right-side BC | | | | | |  |
| --- | --- | --- | --- | --- | --- | --- | --- | --- | --- |
|  |  | Ratio | | Correlation | |  | Liner regression | |  |
|  |  | N/ S | | *r* | *P* value | ***Power (1-β)*** | *R^2^* | *P* value | ***Power (1-β)*** |
| SAN | MHD | 1.89 | | 0.77 | <0.0001 | **99.5%** | 0.47 | <0.0001 | **98.1%** |
|  | LA | 2.89 | | 0.58 | 0.0012 | **80%** | 0.59 | <0.0001 | **>99.9%** |
|  | LV | 3.24 | | 0.61 | 0.001 | **84.4%** | 0.25 | <0.0001 | **61.1%** |
|  | RA | 1.11 | | 0.93 | <0.0001 | **>99.9%** | 0.63 | <0.0001 | **>99.9%** |
|  | RV | 1.23 | | 0.73 | <0.0001 | **98.3%** | 0.46 | <0.0001 | **97.7%** |
| AVN | MHD | 0.72 | | 0.83 | <0.0001 | **>99.9%** | 0.93 | <0.0001 | **>99.9%** |
|  | LA | 1.5 | | 0.59 | 0.0009 | **80.2%** | 0.85 | <0.0001 | **>99.9%** |
|  | LV | 1.57 | | 0.90 | <0.0001 | **>99.9%** | 0.97 | <0.0001 | **>99.9%** |
|  | RA | 0.48 | | 0.65 | 0.0002 | **91.1%** | 0.63 | <0.0001 | **>99.9%** |
|  | RV | 0.55 | | 0.79 | <0.0001 | **99.7%** | 0.90 | <0.0001 | **>99.9%** |

**Correlation analysis post hoc power calculation steps**

1. Select the test type
   1. Open the G*Power software
   2. Select Exact in Test family

1.3 In the Statistical test, select Correlation: Bivariate normal model. The data in this study are non-normally distributed, but because there is no non-normally distributed model for gpower, the approximation can be calculated by Correlation: Bivariate normal model

2. Enter the parameters

Prepare and enter the following parameters for each correlation analysis of (for each row in the table):

2.1 The Tail(s): choose Two (s)

2.2 Alpha err prob (alpha error probability): significance level, this study was corrected for multiple comparisons, using Bonferroni more positive α=0.01

2.3 Total sample size (N): 59 for left analysis and 28 for right analysis

2.4 Correlation ρ H1 (alternative hypothesis correlation ρ): In post hoc analysis, this should be the correlation coefficient r calculated from the study

3. Calculate and read power

3.1 Ensure that the Correlation ρ H0 (the null hypothesis correlation ρ) is 0

3.2 Click the Calculate button in the upper right corner

3.3 The value shown in Power (1-β err prob) in the result box is the statistical power

**linear regression was used to analyze post hoc power calculation steps**

1. Select the test type

1.1 Open the G*Power software

1.2 Select F tests in Test family

1.3 In the Statistical test, select: Linear multiple regression: Fixed model, R² deviation from zero.

2. Enter the parameters

2.1 Effect size f²: This is the most critical parameter. It's calculated by the R squared value. α err prob (alpha error probability): significance level. It is usually set to 0.05. In this study, Bonferroni was used to compare the positive α=0.01.

2.2 Total sample size (N): 59 for left analysis and 28 for right analysis.

2.3 Number of predictors: the total number of independent variables in the input model. Simple linear regression, this number is 1.

3. Calculate the effect size f²

3.1 Click the Determine => button next to the Effect size f² input box.

3.2 A new window will appear. In the Squared multiple correlation ρ² field, the R² value calculated from the study is entered.

3.3 Click the Calculate button.

3.4 The software will automatically fill in the calculated value in the Effect size f² field in the main window.

3.5 Click Calculate and transfer to main window to send the effect size back to the main window.

4. Calculate and read power

4.1 Return to the main window and click the Calculate button.

4.2 The value shown in Power (1-β err prob) in the result box is the statistical power.

**Residual analysis of linear correlation regression analysis between SAN and RA dose in left-sided breast cancer**


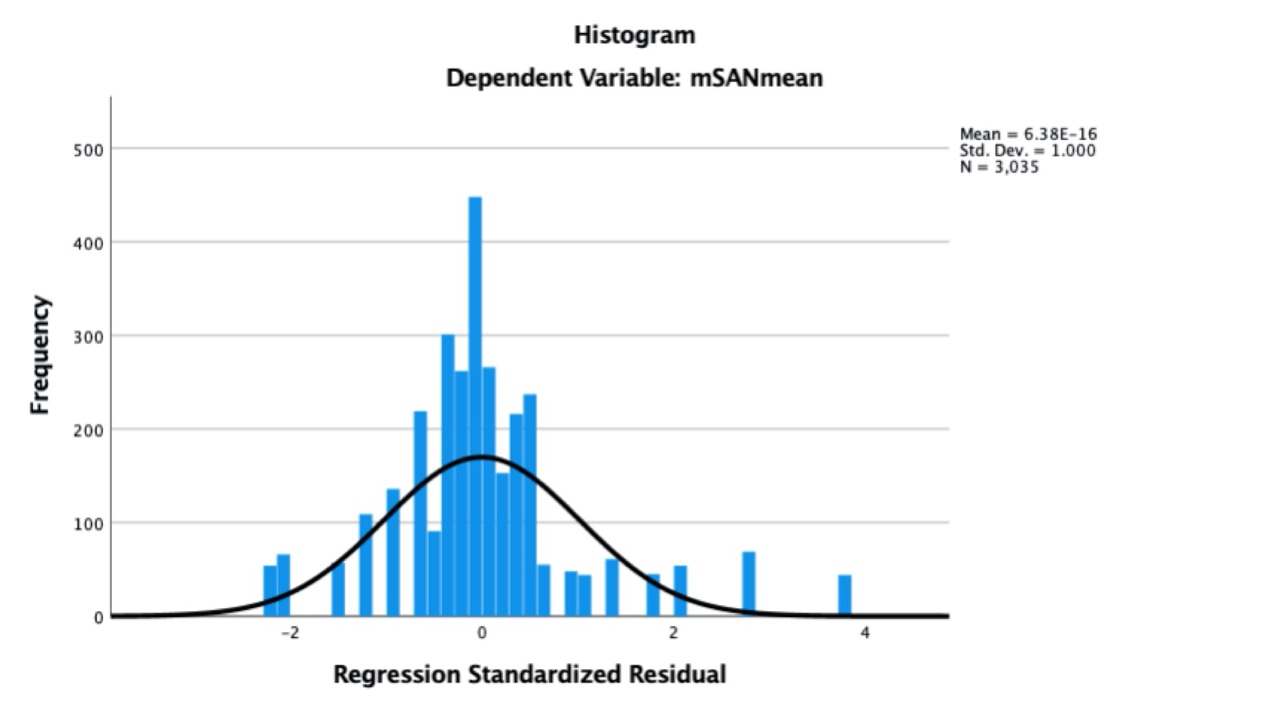


Supplementary Figure 5. Histogram of regression Standardized residuals

The normality of the residuals was judged and showed a normal distribution


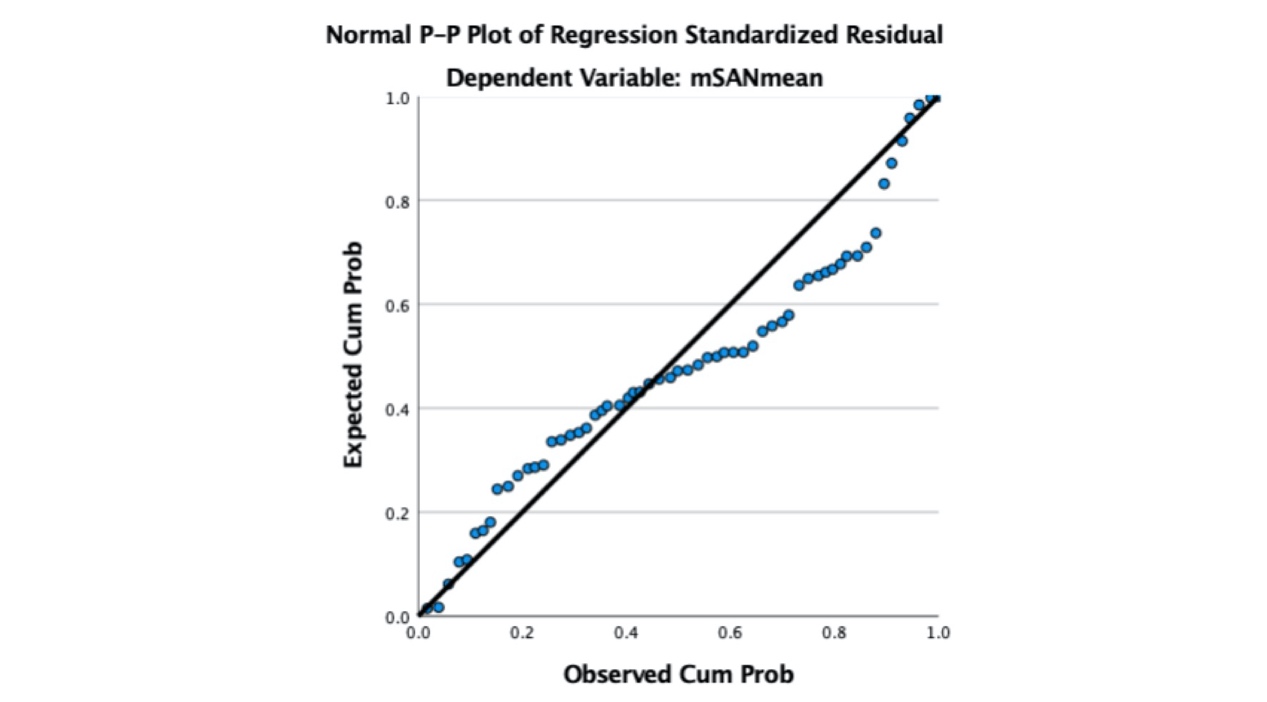


Supplementary Figure 6. Normal P-P plot of regression standardized residuals

It is suggested that the residuals follow approximately a normal distribution


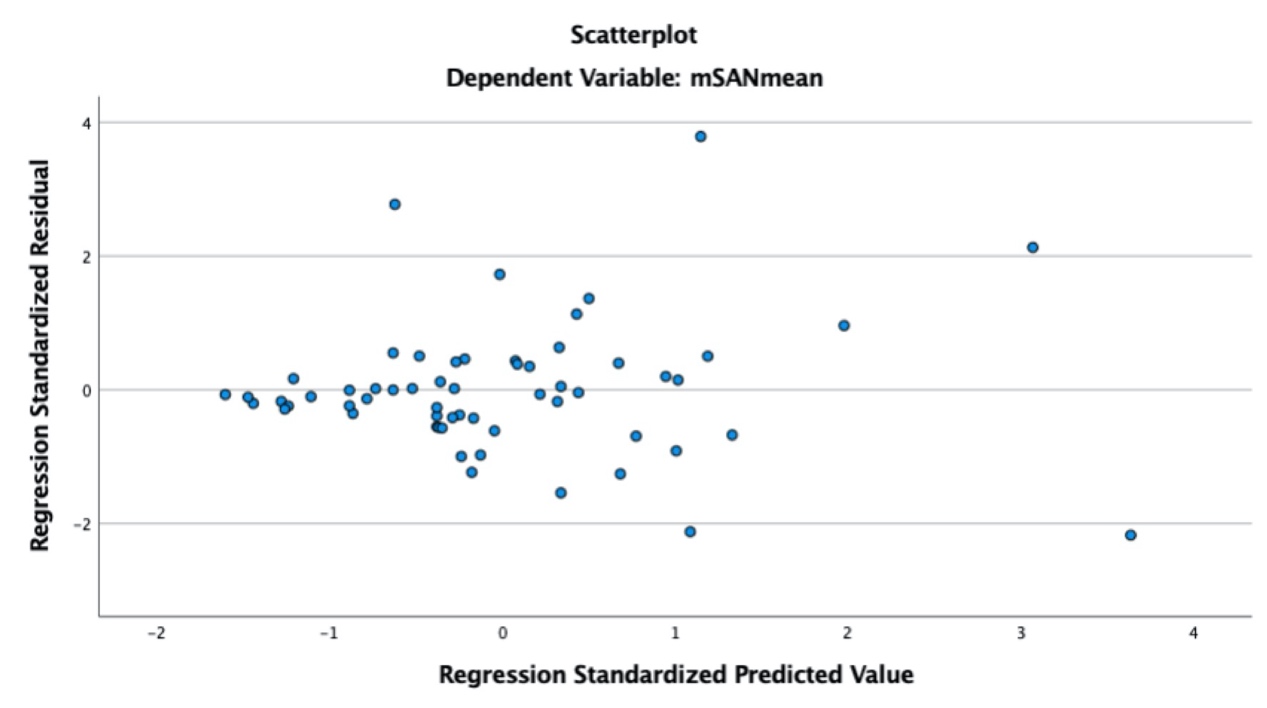


Supplementary Figure 7. Scatter Plot of Standardized Residuals

The predicted value and the corresponding residual were distributed uniformly, and no special distribution form appeared, suggesting that the variance of the residual was homogeneous


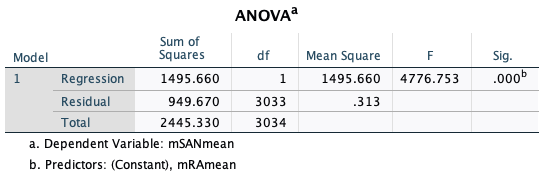


Supplementary Figure 8. Model Statistical test results, using ANOVA, showed that the regression model was statistically significant

**Residual analysis of linear correlation regression analysis between SAN and RA dose in right breast cancer**


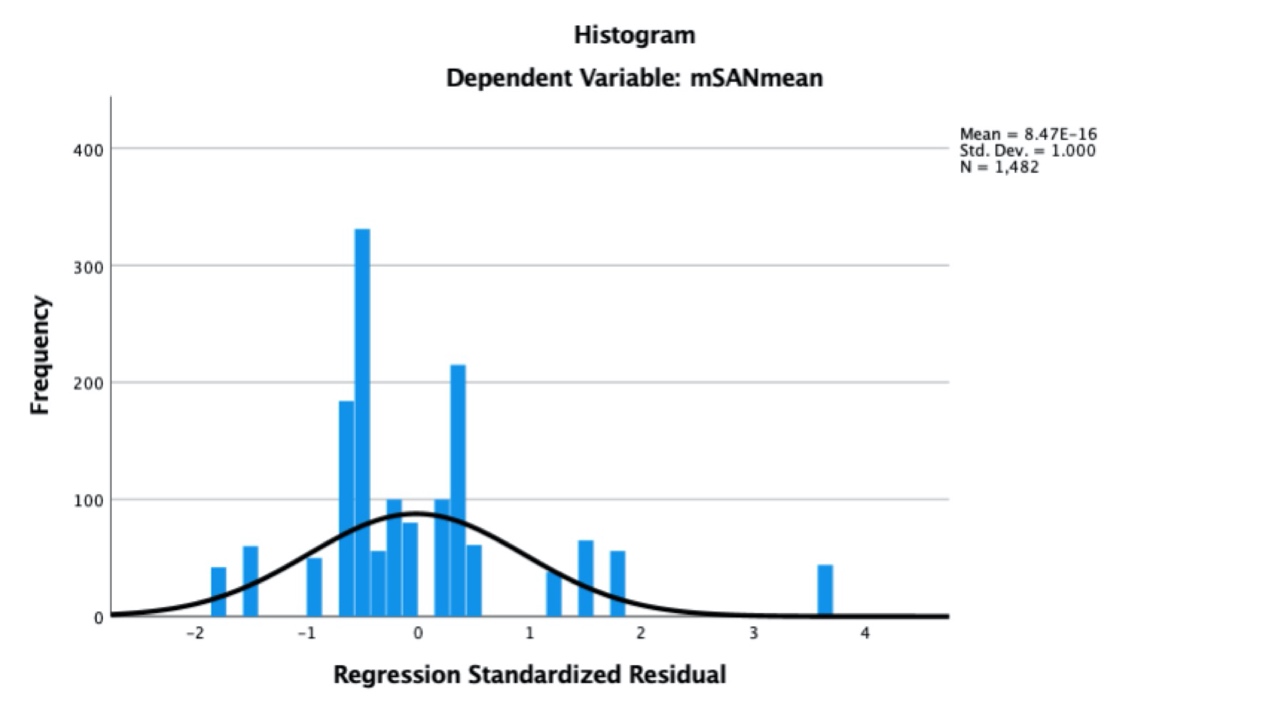


Supplementary Figure 9. Histogram of regression standardized residuals

The normality of the residuals was judged and showed a normal distribution


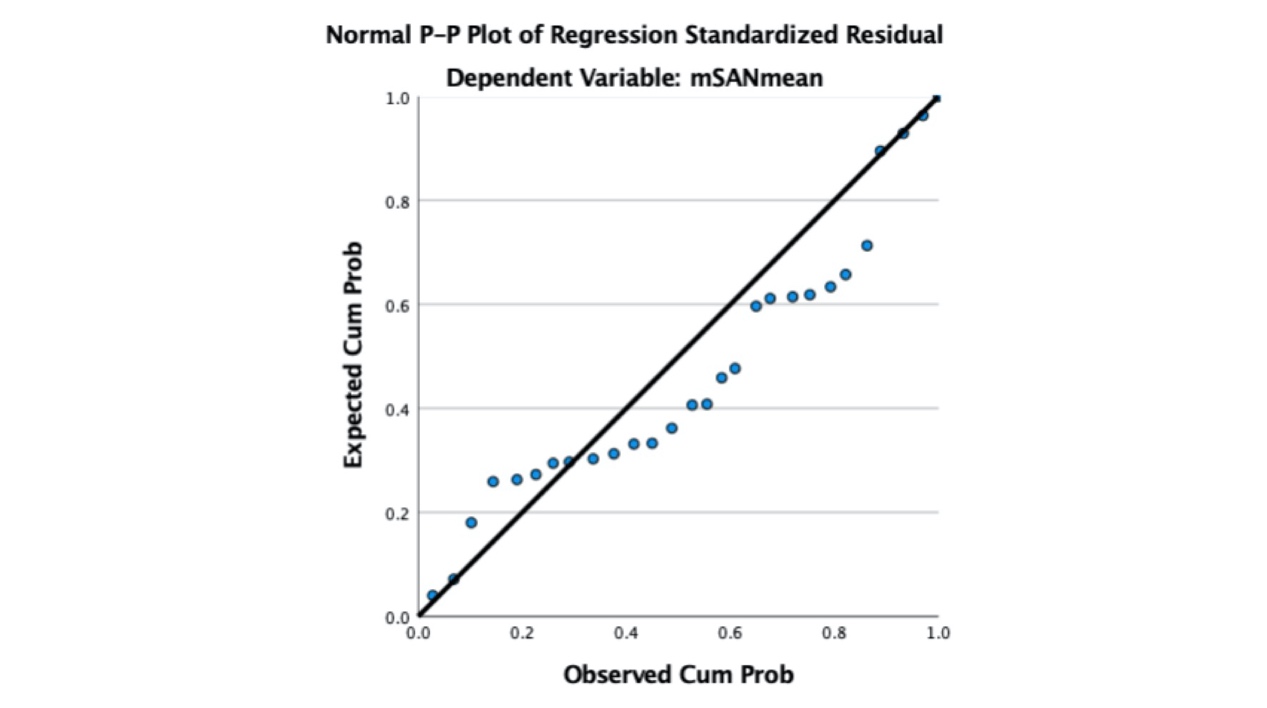


Supplementary Figure 10. Normal P-P plot of regression standardized residuals

It is suggested that the residuals follow approximately a normal distribution


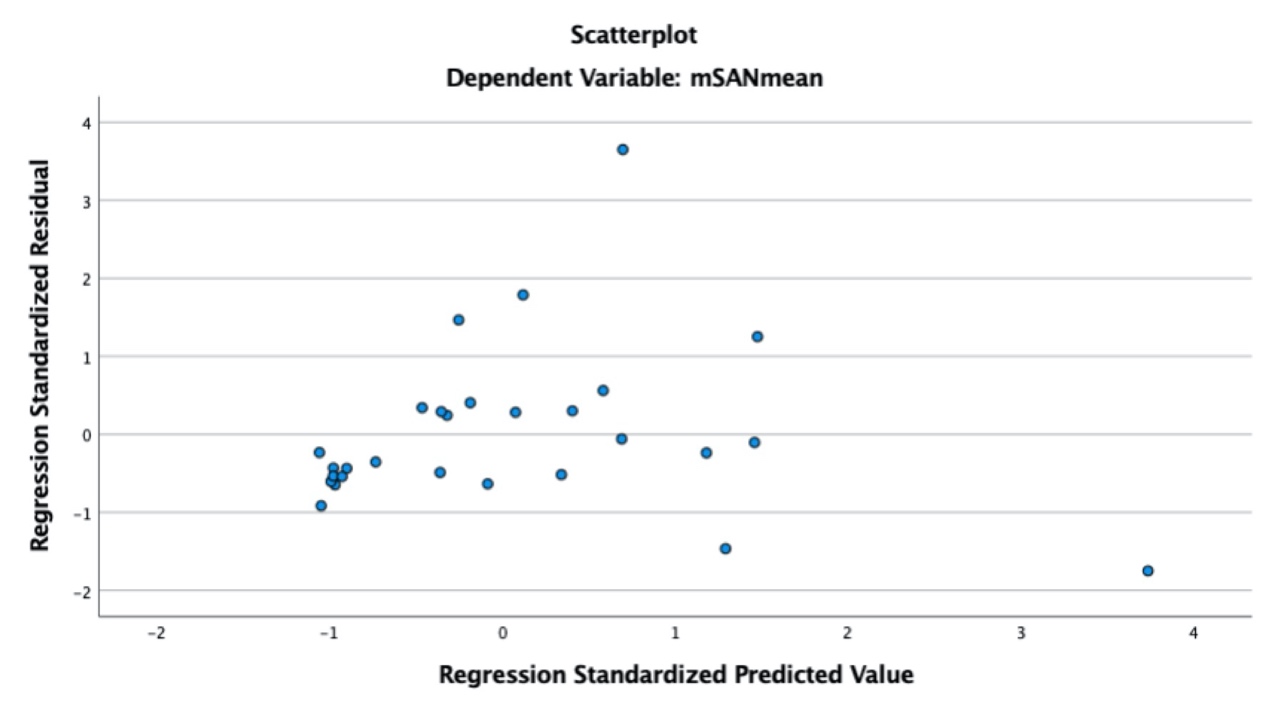


Supplementary Figure 11. Scatter Plot of Standardized Residuals

The predicted value and the corresponding residual were distributed uniformly, and no special distribution form appeared, suggesting that the variance of the residual was homogeneous


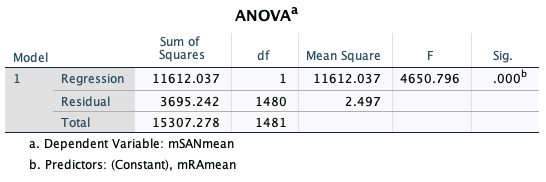


Supplementary Figure 12. Model Statistical test results, using ANOVA, showed that the regression model was statistically significant
